# Backbone phylogeny and adaptive evolution of *Pleurospermum* s. l.: New insights from phylogenomic analyses of complete plastome data

**DOI:** 10.3389/fpls.2023.1148303

**Published:** 2023-03-30

**Authors:** Chang Peng, Xian-Lin Guo, Song-Dong Zhou, Xing-Jin He

**Affiliations:** Key Laboratory of Bio-Resource and Eco-Environment of Ministry of Education, College of Life Sciences, Sichuan University, Chengdu, China

**Keywords:** *Pleurospermum* s. l., Apioideae, complete plastomes, phylogeny, polyphyly

## Abstract

*Pleurospermum* is a taxonomically challenging taxon of Apiaceae, as its circumscription and composition remain controversial for morphological similarities with several related genera, leading to a dispute between *Pleurospermum* in the broad sense and strict sense. While evidence from previous molecular studies recognized plural branching lineages within the *Pleurospermum* s. l., it did not support the latest delimitation of *Pleurospermum* s. str. by only two closely related northern species. So far, no proper delimitation for *Pleurospermum* has come up, and many of the plural taxa in *Pleurospermum* s. l. remain unresolved, which may be due to poor phylogenetic resolution yielded barely from ITS sequences. Herein, we newly assembled 40 complete plastomes from 36 species of *Pleurospermum* s. l. and related genera, 34 of which were first reported and generated a well-resolved backbone phylogeny in a framework of the subfamily Apioideae. From the phylogeny with greatly improved resolution, a total of six well-supported monophyletic lineages within *Pleurospermum* s. l. were recognized falling in different major clades of Apioideae. Combining morphological characteristics with phylogenetic inference, we suggested to re-delimit the *Pleurospermum* s. str. by introducing nine species mainly from the Himalayan regions and proposed its boundary features; the remaining species were suggested to be excluded from *Pleurospermum* to incorporate into their more related taxa being revealed. On this basis, the plastome comparison revealed not only the high conservatism but also the mild differences among lineages in plastome structure and gene evolution. Overall, our study provided a backbone phylogeny essential for further studies of the taxonomically difficult taxa within *Pleurospermum* s. l.

## 1 Introduction

The genus *Pleurospermum* Hoffm. belonging to the tribe Pleurospermeae, subfamily Apioideae, is one of the most problematic genera of Apiaceae for both its monophyly and species composition. It contains ca. 50 mainly alpine herbs with a wide distribution in Eastern Europe and northern Asia and is especially diverse in the Himalayan region and west of China (Pan and Watson, 2005; Pimenov, 2017). Many of its members are used as ethnic herbal medicine by residents. Established in 1816, the original *Pleurospermum* consisted of only three species, *P. austriacum* Hoffm., *P. uralense* Hoffm., and *P. camtschaticum* Hoffm. Unlike those famous members, such as *P. amabile* Craib & W. W. Smith and *P. davidii* Franch., for their remarkable pseudanthia composed of membranous-margined bracts and bracteoles that are distributed in the Himalayan region, these three original members are actually much less developed in bracts and bracteoles. Instead, this genus was established with an emphasis on their developed fruit ribs, which is indicated by its genus name. These three species are considered very closely related, and *P. camtschaticum* has been treated as a synonym of *P. uralense* (Pimenov and Kljuykov, 2000a; Pan and Watson, 2005; Pimenov, 2017), while *P. austriacum* and *P. uralense* are sometimes regarded as two subspecies of a single species (af Rantzien, 1946). They constituted the subgen. *Eupleurospermum* or subgen. *Pleurospermum* in some intrageneric classification and were referred to as “northern” species by some studies because of their distribution in lower altitude regions of Europe, Siberia, and the Far East, which is the northern part of the range of *Pleurospermum* s. l. (Hoffmann, 1816). By contrast, the majority of species commonly treated under *Pleurospermum* are distributed in the southern part of the range, centering in the Sino-Himalayan floristic region, and were discovered, described in, or transferred to *Pleurospermum* by plant hunters exploring these regions. These species are more morphologically diverse, resulting in the complex and controversial taxonomy of this genus.

Based on different opinions, contradictory treatment was applied to *Pleurospermum*, where species or genera with similar morphological characteristics were merged into or segregated from *Pleurospermum* by different authors (Drude, 1897-1898; Farille et al., 1985; Pimenov and Kljuykov, 2000a; Pimenov and Kljuykov, 2000b; Pimenov et al., 2000). With multiple memberships changed, the diagnostic character of *Pleurospermum* and related genera varied, leaving them to have blurred generic boundaries. These related genera include *Hymenidium* DC., *Hymenolaena* DC., and *Pterocyclus* Klotzsch. that are treated as synonyms of *Pleurospermum* in most literature and *Aulacospermum* Ledeb., *Physospermopsis* H. Wolff, and *Trachydium* Lindl. that share some disputed species with *Pleurospermum.* Delimitation of *Pleurospermum* s. str. was carried out by Pimenov et al. in the most recent but radical taxonomic revision of *Pleurospermum* (Pimenov and Kljuykov, 2000a; Pimenov and Kljuykov, 2000b; Pimenov et al., 2000). Based on 47 morphological characteristics of 80 species from *Pleurospermum* and its related genera, they excluded most species from *Pleurospermum* and attributed them to *Aulacospermum*, *Hymenidium*, *Hymenolaena*, *Physospermopsis*, and *Pterocyclus.* The *Pleurospermum* s. str. was then delimited by only two northern species (*P. austriacum* and *P. uralense*), while the *Hymenidium* they restored received those disputed taxa. However, this revision has not been widely accepted, and the broad sense version of *Pleurospermum*, which was referred to as *Pleurospermum* s. l. in later studies, is still widely used. For example, in the *Flora of China* (FOC), the latest regional floras where two-thirds of *Pleurospermum* and more than half of *Trachydium* and *Physospermopsis* species are distributed in, the authors still describe *Pleurospermum* as a genus containing about 50 species with *Aulacospermum*, *Hymenidium*, *Hymenolaena*, and *Pterocyclus* treated as its synonyms (Pan and Watson, 2005). Meanwhile, taxonomists of both sides admitted that neither the traditional *Pleurospermum* (*Pleurospermum* s. l.) nor the restored *Hymenidium* is monophyletic, containing probably some more natural species groups within. Moreover, this has been confirmed by molecular evidence in later studies.

In 2012, an important molecular study of *Pleurospermum* s. l. using nrDNA internal transcript spacer (ITS) and several cpDNA sequences from 55 *Pleurospermum* s. l. and related genera was published (Valiejo-Roman et al., 2012). The results revealed the broad polyphyly in *Pleurospermum* s. l. as the investigated species clustered into three well-supported clades that can be further divided into 11 subclades and scattered in the subfamily Apioideae. Combined with the major clades of Apioideae described by Downie et al. (2010) and some molecular analyses of studies involving *Pleurospermum* species (Zhou et al., 2008; Zhou et al., 2009; Guo M. et al., 2020; Guo X-L. et al., 2020; Zhou et al., 2020a; Xu et al., 2021; Zhou et al., 2021), these lineages within *Pleurospermum* s. l. can be assigned to at least six major clades: Pleurospermeae, *Acronema* clade, East Asia clade, *Komarovia* clade, *Sinodielsia* clade, and *Pleurospermopsis* clade. Meanwhile, the subclade comprised of two northern species of *Pleurospermum* s. str. was found sister to a subclade composed of six southern species that were assigned to *Hymenidium* based on morphology, and the nomenclatural type of *Hymenidium*, *H. brunonis*, is far from them. Therefore, the present form of both *Pleurospermum* in the broad sense and *Pleurospermum* s. str. by segregating the southern species is not supported by molecular data. Since traditional views of taxonomy were conflicting and failed to provide sufficient clues to proper taxonomic revision for *Pleurospermum*, evidence from the microscopic level has been given weight. Some of the lineages within *Pleurospermum* s. l. have recently been revised on the basis of molecular data, including the segregation of *Pterocyclus* (Zhou et al., 2021) and *Pleurospermopsis* (Zhou et al., 2020a). However, here comes another problem: previous phylogenetic analyses are based barely on the commonly used markers such as ITS and a few plastid fragments. Limited loci provided by these datasets yielded phylogeny with poor resolution, especially at lower taxonomic levels. The weakly supported and conflicting tree topology thus hindered the proper taxonomy revision and further understanding of the diversification of *Pleurospermum* s. l. and its related genera.

Herein, according to an updated checklist (Pimenov, 2017) and the *Flora of China* (Pan and Watson, 2005), we obtained plastome data from 40 samples of 36 species, consisting of 29 species from *Pleurospermum* s. l. (including *Pleurospermum* s. str., *Hymenidium*, and *Pterocyclus*) and 7 from its related genera (*Aulacospermum*, *Physospermopsis*). As all samples we collected are from China, we adopted the scientific names from the FOC for most species in this paper for convenience except for those revised in our previous studies. A comparison of their accepted name in different studies and their position indicated from previous molecular studies is listed in **Table 1**. Phylogenetic analyses in the framework of Apioideae and comparative plastome analyses as well as molecular evolutionary analysis were then conducted on the basis of phylogenetic inference. Our aims were to 1) verify the heterogeneity of *Pleurospermum* s. l. and figure out the phylogenetic relationships of its plural lineages; 2) explore the species composition of the real *Pleurospermum* and the proper taxonomic boundaries between *Pleurospermum* and its related genera, as well as the proper taxonomic position of the other species of *Pleurospermum* s. l.; and 3) infer the diversity of plastome characteristics and structural evolution and adaptation of these taxa. Overall, our results will lay the foundation for further phylogenetic and taxonomic studies of *Pleurospermum* s. l.

**Table 1:** Accepted name of the 36 species in different literature and their taxonomic position.

Accepted name in this study	Basionym	Accepted name in the latest checklist by Pimenov et al.	Accepted name in the *Flora of China*	Position indicated by a previous molecular study
*Pleurospermum giraldii* Diels	*Pleurospermum giraldii* Diels	*Hymenidium giraldii* (Diels) Pimenov & Kljuykov	*Pleurospermum giraldii* Diels	*Acronema* clade
*Pleurospermum hookeri* C.B. Clarke	*Pleurospermum hookeri* C.B. Clarke	*Hymenidium hookeri* (C.B. Clarke) Pimenov & Kljuykov	*Pleurospermum hookeri* C.B. Clarke	*Acronema* clade
*Pleurospermum yunnanense* Franch.	*Pleurospermum yunnanense* Franch.	*Hymenidium yunnanense* (Franch.) Pimenov & Kljuykov	*Pleurospermum yunnanense* Franch.	*Acronema* clade
*Pleurospermum amabile* Craib & W.W. Sm.	*Pleurospermum amabile* Craib & W.W. Sm.	*Hymenidium amabile* (Craib & W.W. Sm.) Pimenov & Kljuykov	*Pleurospermum amabile* Craib & W.W. Sm.	East Asia clade
*Pleurospermum angelicoides* (DC.) Benth. ex C.B. Clarke	*Hymenolaena angelicoides* DC.	*Pterocyclus angelicoides* (DC.) Klotzsch	*Pleurospermum angelicoides* (DC.) Benth. ex C.B. Clarke	*Komarovia* clade
*Pleurospermum rotundatum* (DC.) C.B. Clarke	*Hymenolaena rotundata* DC.	*Pterocyclus rotundatus* (DC.) Pimenov & Kljuykov	*Pleurospermum rotundatum* (DC.) C.B. Clarke	*Komarovia* clade
*Pterocyclus forrestii* (Diels) Pimenov & Kljuykov	Angelica forrestii Diels	*Pterocyclus forrestii* (Diels) Pimenov & Kljuykov	*Pleurospermum angelicoides* (Wall.) Benth. ex C. B. Clarke	*Komarovia* clade
*Aulacospermum anomalum* Ledeb.	*Aulacospermum anomalum* Ledeb.	*Aulacospermum anomalum* Ledeb.	/	Pleurospermeae
*Physospermopsis delavayi* (Franch.) H. Wolff	*Arracacia delavayi* Franch.	*Physospermopsis delavayi* (Franch.) H. Wolff	*Physospermopsis delavayi* (Franch.) H. Wolff	Pleurospermeae
*Physospermopsis kingdon-wardii* (H. Wolff) C. Norman	*Trachydium kingdon-wardii* H. Wolff	*Physospermopsis obtusiuscula* (DC.) C. Norman	*Physospermopsis kingdon-wardii* (H. Wolff) C. Norman	Pleurospermeae
*Physospermopsis muliensis* R.H. Shan & S.L. Liou	*Physospermopsis muliensis* R.H. Shan & S.L. Liou	*Physospermopsis muliensis* R.H. Shan & S.L. Liou	*Physospermopsis muliensi*s R.H. Shan & S.L. Liou	Pleurospermeae
*Physospermopsis nana* (Franch.) Pimenov & Kljuykov	*Pleurospermum nanum* Franch.	*Physospermopsis nana* (Franch.) Pimenov & Kljuykov	*Pleurospermum nanum* Franch.	Pleurospermeae
*Physospermopsis obtusiuscula* (DC.) C. Norman	*Hymenolaena obtusiuscula* DC.	*Physospermopsis obtusiuscula* (DC.) C. Norman	*Physospermopsis obtusiuscula* (DC.) C. Norman	Pleurospermeae
*Physospermopsis rubrinervis* (Franch.) C. Norman	*Trachydium rubrinerve* Franch.	*Physospermopsis rubrinervis* (Franch.) C. Norman	*Physospermopsis rubrinervis* (Franch.) C. Norman	Pleurospermeae
*Physospermopsis shaniana* C.Y. Wu & F.T. Pu	*Physospermopsis shaniana* C.Y. Wu & F.T. Pu	*Physospermopsis shaniana* C.Y. Wu & F.T. Pu	*Physospermopsis shaniana* C.Y. Wu & F.T. Pu	Pleurospermeae
*Pleurospermum benthamii* (DC.) C.B. Clarke	*Hymenolaena benthamii* DC.	*Hymenidium benthamii* (DC.) Pimenov & Kljuykov	/	Pleurospermeae
*Pleurospermum cristatum* H. Boissieu	*Pleurospermum cristatum* H. Boissieu	*Hymenidium cristatum* (H. Boissieu) Pimenov & Kljuykov	*Pleurospermum cristatum* H. Boissieu	Pleurospermeae
*Pleurospermum davidii* Franch.	*Pleurospermum davidii* Franch.	*Hymenidium davidii* (Franch.) Pimenov & Kljuykov	*Pleurospermum benthamii* (DC.) C.B. Clarke	Pleurospermeae
*Pleurospermum decurrens* Franch.	*Pleurospermum decurrens* Franch.	*Hymenidium decurrens* (Franch.) Pimenov & Kljuykov	*Pleurospermum decurrens* Franch.	Pleurospermeae
*Pleurospermum foetens* Franch.	*Pleurospermum foetens* Franch.	*Hymenidium foetens* (Franch.) Pimenov & Kljuykov	*Pleurospermum foetens* Franch.	Pleurospermeae
*Pleurospermum hedinii* Diels	*Pleurospermum hedinii* Diels	*Hymenidium hedinii* (Diels) Pimenov & Kljuykov	*Pleurospermum hedinii* Diels	Pleurospermeae
*Pleurospermum linearilobum* W.W. Sm.	*Pleurospermum linearilobum* W.W. Sm.	*Hymenidium linearilobum* (W.W. Sm.) Pimenov & Kljuykov	*Pleurospermum linearilobum* W.W. Sm.	Pleurospermeae
*Pleurospermum szechenyii* Kanitz	*Pleurospermum szechenyii* Kanitz	*Hymenidium szechenyii* (Kanitz) Pimenov & Kljuykov	*Pleurospermum szechenyii* Kanitz	Pleurospermeae
*Pleurospermum uralense* Hoffm.	*Pleurospermum uralense* Hoffm.	*Pleurospermum uralense* Hoffm.	*Pleurospermum uralense* Hoffm.	Pleurospermeae
*Pleurospermum wilsonii* H. Boissieu	*Pleurospermum wilsonii* H. Boissieu	*Hymenidium wilsonii* (H. Boissieu) Pimenov & Kljuykov	*Pleurospermum wilsonii* H. Boissieu	Pleurospermeae
*Pleurospermum wrightianum* H. Boissieu	*Pleurospermum wrightianum* H. Boissieu	*Hymenidium wrightianum* (H. Boissieu) Pimenov & Kljuykov	*Pleurospermum wrightianum* H. Boissieu	Pleurospermeae
*Pleurospermum bicolor* (Franch.) C. Norman ex Z.H. Pan & M.F. Watson	*Pleurospermum govanianum* var. *bicolor* Franch.	*Hymenidium bicolor* (Franch.) Pimenov & Kljuykov	*Pleurospermum bicolor* (Franch.) C. Norman ex Z.H. Pan & M.F. Watson	*Pleurospermopsis* clade
*Hymenidium dentatum* (DC.) Pimenov & Kljuykov	*Hymenolaena dentata* DC.	*Hymenidium dentatum* (DC.) Pimenov & Kljuykov	/	*Sinodielsia* clade
*Hymenidium ladyginii* Pimenov et Kljuykov	*Hymenidium ladyginii* Pimenov et Kljuykov	*Hymenidium ladyginii* Pimenov et Kljuykov	/	*Sinodielsia* clade
*Pleurospermum apiolens* C.B. Clarke	*Pleurospermum apiolens* C.B. Clarke	*Hymenidium apiolens* (C.B. Clarke) Pimenov & Kljuykov	*Pleurospermum apiolens* C.B. Clarke	*Sinodielsia* clade
*Pleurospermum aromaticum* W.W. Sm.	*Pleurospermum aromaticum* W.W. Sm.	*Oreocomopsis dochenensis* (W. W. Sm.) Pimenov & Kljuykov	*Pleurospermum aromaticum* W.W. Sm.	*Sinodielsia* clade
*Pleurospermum rivulorum* (Diels) M. Hiroe	*Angelica rivulorum* Diels	*Pterocyclus rivulorum* (Diels) H. Wolff	*Pleurospermum rivulorum* (Diels) M. Hiroe	*Sinodielsia* clade
*Hymenidium pachycaule* Pimenov & Kljuykov	*Hymenidium pachycaule* Pimenov & Kljuykov	*Hymenidium pachycaule* Pimenov & Kljuykov	/	/
*Pleurospermum heracleifolium* Franch. ex H. Boissieu	*Pleurospermum heracleifolium* Franch. ex H. Boissieu	*Hymenidium heracleifolium* (Franch. ex H. Boissieu) Pimenov & Kljuykov	*Pleurospermum heracleifolium* Franch. ex H. Boissieu	/
*Pleurospermum pulszkyi* Kanitz	*Pleurospermum pulszkyi* Kanitz	*Hymenidium pulszkyi* (Kanitz) Pimenov & Kljuykov	*Pleurospermum pulszkyi* Kanitz	/
*Pleurospermum tsekuense* R.H. Shan	*Pleurospermum tsekuense* R.H. Shan	*Hymenidium tsekuense* (R.H. Shan) Pimenov & Kljuykov	*Pleurospermum tsekuense* R.H. Shan	/

## 2 Materials and methods

### 2.1 Materials, DNA extraction, plastome sequencing, assembly, and annotation

Forty samples from 36 species of *Pleurospermum* s. l. and related genera were obtained from NW and SW China (**Supplementary Table S1**). Fresh, healthy leaves were collected from adult plants on the field and immediately stored in silica gel. Total genomic DNA was extracted from silica-dried leaves with a modified CTAB protocol (Doyle and Doyle, 1987). Voucher specimens were deposited in the herbarium of Sichuan University (SZ), Chengdu, China.

Complete plastomes of the 40 samples were sequenced from an Illumina HiSeq X Ten platform (paired-end, 150 bp) at Novogene (Tianjin, China). Quality control of the raw reads was performed using fastP version v0.15.0 (-n 10 and -q 15), yielding at least 5 GB clean reads for each species. The read quality of clean reads was assessed using FastQC v0.11.9 (Andrews, 2010). *De novo* assemblies were conducted using GetOrganelle v1.7.3.1 (Jin et al., 2020) with the recommended parameters of chloroplast and ribosomal RNA (18S-ITS1-5.8S-ITS2-28S) sequence. To confirm the credibility of the assemblies, another assembly for each material was also performed using NOVOPlasty v2.7.2 (Dierckxsens et al., 2017), with *rbcL* sequence extracted from *P. uralense* (NC_033343) as seed and default parameters. The clean reads were mapped to the draft genome using Geneious v9 (Kearse et al., 2012) to check the concatenation of contigs. The annotation was conducted firstly by PGA (Qu et al., 2019) using *P. uralense* (NC_033343) as reference. The start and stop codons and intron positions were manually corrected according to the plastomes of congeneric species in Geneious. The annotation of tRNAs and rRNAs was verified by Infernal with the Rfam database (Kalvari et al., 2018; Kalvari et al., 2021). The gene map was drawn using the online program OrganellarGenomeDRAW (OGDRAW) (Greiner et al., 2019).

### 2.2 Phylogenetic analyses

To infer the phylogenetic relationships of these 36 species from *Pleurospermum* s. l. and related genera in a framework of Apioideae, we conducted plastid phylogenetic reconstruction using plastomes from all over the Apioideae as representatives of major clades of Apioideae described by Downie et al (Zhou et al., 2020a). Forty-nine plastomes of 40 genera (listed in **Supplementary Table S2**) were chosen to represent 17 major clades, and two plastomes from *Sanicula* were chosen as an outgroup. All these plastomes were downloaded from NCBI (https://www.ncbi.nlm.nih.gov/) and reannotated using the calibrated annotation of *P. uralense* (NC_033343) as reference. The 79-PCG (protein-coding genes) dataset from the 91 plastomes in total was extracted by PhyloSuite v1.2.2 (Zhang et al., 2020), and then aligned by MAFFT (Katoh and Standley, 2013), trimmed by trimAl (Capella-Gutierrez et al., 2009) with no gaps option, and concatenated by PhyloSuite with default settings. Phylogenetic analysis was performed using both maximum likelihood (ML) and Bayesian inference (BI) methods. ModelFinder (Kalyaanamoorthy et al., 2017) was used to select the best-fit partition model (Edge-unlinked) using the corrected Akaike information criterion (AICc) for IQ-TREE and MrBayes. The ML phylogenies were inferred using IQ-TREE (Nguyen et al., 2015) under the Edge-linked partition model for 5,000 ultrafast bootstraps and Shimodaira–Hasegawa-like approximate likelihood-ratio test. BI analyses were conducted using MrBayes 3.2.6 (Ronquist et al., 2012) under the partition model (two parallel runs, 10,000,000 generations), in which the initial 25% of sampled data were discarded as burn-in.

Phylogenetic analysis of the nrDNA ITS dataset was also conducted for comparison. Seventy-seven sets of ITS1+ITS2 from 76 species corresponding to species or genus applied in plastid phylogenetic reconstruction were obtained from NCBI for phylogenetic inference, and 43 of them are related to *Pleurospermum* s. l. ML and BI analyses were conducted following the workflow above. Also, as the available plastomes from Apioideae are limited, we conducted an expanded analysis adding an extra 91 sets of ITS1+ITS2 from NCBI for an expanded phylogenetic reconstruction to cover more major clades of Apioideae and species related to *Pleurospermum* s. l. All the sequences used and the major clades they represent are listed in **Supplementary Table S2**.

### 2.3 Plastome comparative analyses

Based on phylogenetic inference, we performed several comparative analyses among the 40 newly assembled plastomes and 3 downloaded plastomes from *Pleurospermum* s. l. (*P. uralense*, *P. amabile*, and *Ligusticum delavayi*) that were also used in the phylogenetic analyses. The length and gene of each plastome were identified, and the guanine-cytosine (GC) content of each region was calculated with Geneious v9 (Kearse et al., 2012). To detect the presence of IR expansion or contraction, all plastomes were aligned and compared by the MAFFT multiple aligner plugin of Geneious. The DNA rearrangements among these plastomes were also detected by Mauve Alignment (Darling et al., 2010) implemented in Geneious. For identifying hypervariable regions, the whole plastome alignment of the 43 plastomes was visualized using the mVISTA with Shuffle-LAGAN mode (Frazer et al., 2004), and annotation of *P. uralense* (NC_033343) was taken as a reference. All protein-coding and non-coding regions (intergeneric spacers and intron) over 200 bp were extracted and aligned in PhyloSuite, and their nucleotide diversity (Pi) values were calculated by DnaSP 6 (Rozas et al., 2017) to detect genetic divergence.

Seventy-nine PCGs shared by the 43 plastomes were extracted for codon analysis. Codon usage and relative synonymous codon usage (RSCU) values were calculated using DnaSP 6. The level of codon usage bias was determined by calculating ENC, CBI, GC2, and GC3. The RSCU distribution was illustrated in the form of heatmaps using TBtools (Chen et al., 2018). The base compositions for all PCGs were calculated by MEGA X (Kumar et al., 2018).

### 2.4 RNA edit and selective pressure analysis

The online program Predictive RNA Editors for Plants (PREP) suite (Mower, 2009) was used to predict the possible RNA editing sites in all 35 genes available, based on a cutoff value of 0.8 (optimal for PREP-Cp). The Branch-Site Unrestricted Statistical Test for Episodic Diversification (BUSTED) (Murrell et al., 2015) and adaptive Branch-Site Random Effects Likelihood (aBSREL) (Smith et al., 2015) from HyPhy (v.2.5.32) package were applied to the coding DNA sequence (CDS) of the 76 shared PCGs to test for alignment-wide episodic diversifying selection and find lineages which have experienced episodic diversifying selection. The ML tree generated *via* IQ-TREE was used as a constraint topology, and the six clades revealed by the phylogenetic analysis were set as the foreground branches, respectively. Significance was assessed using the likelihood ratio test at a threshold of *p ≤*0.05 after correcting for multiple testing. Also, the Mixed Effects Model of Evolution (MEME) (Murrell et al., 2012) analysis was also conducted to search the individual sites evolving under positive selection in a proportion of branches.

## 3 Result

### 3.1 Characteristics of the plastomes

Illumina sequencing generated 30,084,236 to 73,066,366 paired-end clean reads for 40 samples of *Pleurospermum* s. l. Among them, 98,817 to 3,851,352 reads were mapped to the final assembly, with coverage ranging from 93.11 X to 3,725.428 X (**Supplementary Table S1**). The plastomes of 34 of these 36 species are first reported here. All the plastomes obtained display a typical quadripartite structure: a large single copy region (LSC, 84,854–101,725 bp), a small single copy region (SSC, 16,751–19,090 bp), and a pair of inverted repeat regions (IRa and IRb, 17,036–28,584 bp) (**Table 2**, **Supplementary Figure S1**). The genome size ranged from 14,682 bp (*P. aromaticum*) to 171,866 bp (*P. giraldii*). The overall GC content is between 37.4% (*P. apiolens*) and 38.1% (*P. davidii*). Comparison among the GC contents of these regions shows that IR regions share the highest percentage (41.7%–45.4%), while SSC regions are the lowest (30.9%–33.6%). Gene contents are conserved as all species contain 113 distinct genes, consisting of 79 protein-coding genes, 30 tRNA, and 4 rRNA genes. Gene contents are listed in **Supplementary Table S3**. The overall gene number is 130 (consisting of 85 PCGs, 37 tRNA genes, and 8 rRNA genes) in most species, but fewer (126 genes) in the four species from group E (*P. apiolens*, *H. dentatum*, *P. rivulorum*, and *P. aromaticum*) as they lose three copies of PCGs (*ycf2*, *rpl2*, and *rpl23*) and one tRNA gene (trnI-CAU) that result from the contraction of IR regions on the LSC/IRa border.

**Table 2:** General features of the 43 plastomes from *Pleurospermum* s. l. and related genera.

Taxa	Length				Genes							GC content			
					In regions (LSC/SSC/IR)			Total							
	Total	LSC	SSC	IR	PCG	tRNAs	rRNAs	PCG	tRNAs	rRNAs	Total	Total	LSC	SSC	IR
*Aulacospermum anomalum*	155,948	85,642	17,808	26,249	63/12/7	22/1/7	0/0/4	85	37	8	130	37.9	36.1	32	42.8
*Pleurospermum hedinii*	156,185	85,655	17,758	26,386	63/12/7	22/1/7	0/0/4	85	37	8	130	37.9	36.1	31.8	42.8
*Pleurospermum uralense*	155,415	85,944	17,493	25,989	63/12/7	22/1/7	0/0/4	85	37	8	130	37.9	36.1	32.2	43
*Pleurospermum cristatum*	155,672	85,498	17,678	26,248	63/12/7	22/1/7	0/0/4	85	37	8	130	38	36.2	32.3	42.9
*Pleurospermum davidii*	155,656	85,772	17,712	26,086	63/12/7	22/1/7	0/0/4	85	37	8	130	38.1	36.3	32.3	42.9
*Pleurospermum decurrens* KM	155,460	85,178	17,738	26,272	63/12/7	22/1/7	0/0/4	85	37	8	130	37.9	36.2	32.2	42.9
*Pleurospermum decurrens* DL	155,503	85,302	17,769	26,216	63/12/7	22/1/7	0/0/4	85	37	8	130	38	36.2	32.2	42.9
*Pleurospermum foetens*	155,298	85,609	17,637	26,026	63/12/7	22/1/7	0/0/4	85	37	8	130	38	36.2	32.3	42.9
*Pleurospermum heracleifolium*	155,580	85,352	17,720	26,254	63/12/7	22/1/7	0/0/4	85	37	8	130	38	36.2	32.1	42.9
*Pleurospermum linearilobum*	155,592	85,778	17,710	26,052	63/12/7	22/1/7	0/0/4	85	37	8	130	38	36.2	32.3	42.9
*Pleurospermum benthamii*	155,924	85,591	17,793	26,270	63/12/7	22/1/7	0/0/4	85	37	8	130	37.9	36.2	32	42.9
*Pleurospermum szechenyii*	156,008	85,737	17,789	26,241	63/12/7	22/1/7	0/0/4	85	37	8	130	37.9	36.1	32	42.9
*Pleurospermum uralense*	155,426	85,965	17,483	25,989	63/12/7	22/1/7	0/0/4	85	37	8	130	37.9	36.1	32.2	43
*Pleurospermum wrightianum*	155,925	85,664	17,795	26,243	63/12/7	22/1/7	0/0/4	85	37	8	130	37.9	36.1	32	42.9
*Physospermopsis kingdon-wardii*	156,166	86,489	17,899	25,889	63/12/7	22/1/7	0/0/4	85	37	8	130	37.9	36.1	32.1	42.9
*Physospermopsis obtusiuscula*	156,161	86,455	17,926	25,890	63/12/7	22/1/7	0/0/4	85	37	8	130	37.9	36.1	32.1	42.9
*Pleurospermum wilsonii* KD	155,936	85,772	17,664	26,275	63/12/7	22/1/7	0/0/4	85	37	8	130	37.9	36.1	32.1	42.9
*Pleurospermum wilsonii* YJ	156,104	85,902	17,650	26,276	63/12/7	22/1/7	0/0/4	85	37	8	130	37.9	36.1	32.1	42.9
*Physospermopsis delavayi* ZD	156,302	86,055	18,037	26,105	63/12/7	22/1/7	0/0/4	85	37	8	130	37.9	36	31.9	43
*Physospermopsis delavayi* LJ	156,222	85,868	18,084	26,135	63/12/7	22/1/7	0/0/4	85	37	8	130	37.9	36.1	31.9	42.9
*Physospermopsis muliensis*	156,376	85,894	17,860	26,311	63/12/7	22/1/7	0/0/4	85	37	8	130	37.9	36.1	32	42.9
*Physospermopsis nana* LP	154,510	84,759	17,835	25,958	63/12/7	22/1/7	0/0/4	85	37	8	130	37.9	36.1	32	42.9
*Physospermopsis nana* DQ	155,194	84,854	17,828	26,256	63/12/7	22/1/7	0/0/4	85	37	8	130	37.9	36.1	32	42.9
*Physospermopsis rubrinervis*	155,942	85,453	17,973	26,258	63/12/7	22/1/7	0/0/4	85	37	8	130	37.9	36	32	42.9
*Physospermopsis shaniana*	156,551	86,054	17,875	26,311	63/12/7	22/1/7	0/0/4	85	37	8	130	37.9	36.1	32	42.9
*Pleurospermum angelicoides*	157,196	86,696	17,982	26,181	63/12/7	22/1/7	0/0/4	85	37	8	130	37.7	35.8	31.4	42.9
*Pterocyclus forrestii*	156,405	86,011	17,898	26,248	63/12/7	22/1/7	0/0/4	85	37	8	130	37.7	35.9	31.5	42.9
*Pleurospermum rotundatum*	156,476	86,012	17,981	26,240	63/12/7	22/1/7	0/0/4	85	37	8	130	37.7	35.8	31.3	42.9
*Pleurospermum amabile*	155,955	85,745	17,562	26,324	63/12/7	22/1/7	0/0/4	85	37	8	130	37.7	35.8	31.4	42.9
*Pleurospermum amabile* DQ	155,922	85,722	17,580	26,310	63/12/7	22/1/7	0/0/4	85	37	8	130	37.6	35.7	31.4	42.9
*Hymenidium pachycaule*	156,293	85,705	17,954	26,317	63/12/7	22/1/7	0/0/4	85	37	8	130	37.6	35.7	31.2	42.9
*Pleurospermum pulszkyi*	155,993	85,629	17,716	26,324	63/12/7	22/1/7	0/0/4	85	37	8	130	37.7	35.8	31.5	42.9
*Pleurospermum tsekuense*	161,932	85,674	19,090	28,584	63/12/7	22/1/7	0/0/4	85	37	8	130	37.6	35.7	33.6	41.7
*Ligusticum delavayi*	155,621	85,064	16,741	26,908	63/12/7	22/1/7	0/0/4	85	37	8	130	37.6	35.7	31	42.5
*Pleurospermum giraldii*	171,866	101,725	17,511	26,315	63/12/7	22/1/7	0/0/4	85	37	8	130	38	36.8	30.9	42.8
*Pleurospermum hookeri*	155,512	84,967	16,787	26,879	63/12/7	22/1/7	0/0/4	85	37	8	130	37.5	35.7	31	42.5
*Hymenidium ladyginii*	155,695	85,374	17,777	26,272	63/12/7	22/1/7	0/0/4	85	37	8	130	37.6	35.7	30.9	42.58
*Pleurospermum yunnanense*	155,631	85,038	16,751	26,921	63/12/7	22/1/7	0/0/4	85	37	8	130	37.6	35.7	31.1	42.5
*Pleurospermum apiolens*	146,554	94,017	17,597	17,470	63/12/4	22/1/7	0/0/4	82	36	8	126	37.4	35.9	30.9	45
*Hymenidium dentatum*	146,783	94,158	17,599	17,513	63/12/4	22/1/6	0/0/4	82	36	8	126	37.5	35.9	31.1	44.9
*Pleurospermum rivulorum*	146,823	93,189	17,544	18,045	63/12/4	22/1/6	0/0/4	82	36	8	126	37.6	36	31.1	44.8
*Pleurospermum aromaticum*	146,167	94,140	17,955	17,036	63/12/4	22/1/6	0/0/4	82	36	8	126	37.6	36	31	45.4
*Pleurospermum bicolor*	160,183	86,200	17,803	28,090	63/12/7	22/1/7	0/0/4	85	37	8	130	37.9	36	31.8	42.7

### 3.2 Phylogenetic analysis

**Figure 1:**
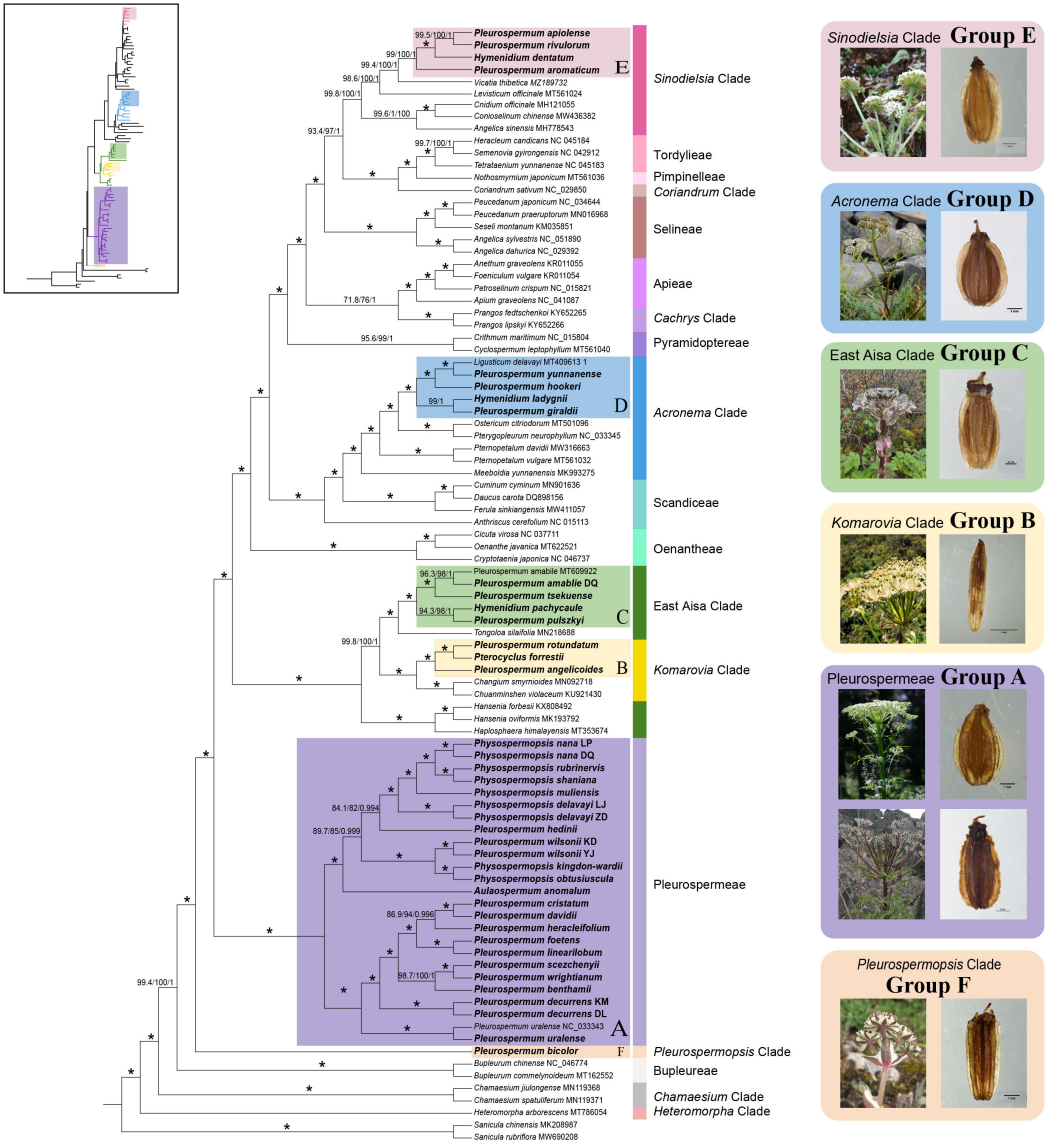
Phylogenetic relationships inferred from 91 plastomes (86 species) based on 79 protein-coding genes (PCGs). The main tree shown here was reconstructed by maximum likelihood (ML). Support values marked above the branches follow the order SH-aLRT support/ultrafast bootstrap support/posterior probability. * represents the best support that the maximum value is for all support rates (100/100/1). The colored strips on the right of the sequence indicate the major clades of Apioideae they belong to; the six recognized lineages are marked with colored ranges. Species selected as representatives of each lineage with inflorescence and fruit shown in boxes on the right are as follows: group A, *P. uralense* and *P. davidii*; group B, *P. angelicoides*; group C, *P. amabile*; group D, *P. hookeri*; group E, *P. apiolens*; group F, *P. bicolor*.

The final alignment of the 79-PCGs from the 91-sample dataset consists of 77,297 columns with 10,624 parsimony-informative sites. The ITS1+ITS2 dataset from 131 samples and the expanded ITS dataset from 207 samples consist of 399 columns with 310 parsimony-informative sites and 396 columns with 321 parsimony-informative sites, respectively. In all trees yielded, major clades of Apioideae represented by several species showed relationships and overall topologies consistent with previous molecular studies, thus forming a reliable framework for phylogenetic inference (**Figure 1**, **Supplementary Figures S2**, **S3**).

In the result of plastome phylogenetic reconstruction, the sequence from *Pleurospermum* s. l. all clearly clustered into six groups and fell into different major clades of Apioideae as follows: group A consisted of 20 species from *Aulacospermum*, *Physospermopsis*, and *Pleurospermum* s. l. and represented the Tribe Pleurospermeae. Nine Himalayan species of *Pleurospermum* s. l. formed a well-supported subclade with *P. uralense* that represented the *Pleurospermum* s. str. The rest of the species from *Aulacospermum* and *Physospermopsis* and two from *Pleurospermum* s. l. formed another subclade. Group B consisted of *P. angelicoides*, *Pt. forrestii*, and *P. rotundatum*, which were treated in *Pterocyclus* in recent studies, and it clustered with *Changium* and *Chuanminshen* that represented the *Komarovia* clade. Group C consisted of four species, *P. amabile*, *P. tsekuense*, *P. pulszkyi*, and *H. pachycaule*, and nested in the East Asia clade. Group D consisted of *P. hookeri*, *P. yunnanense*, *P. giraldii*, and *H. ladyginii* and nested in the *Acronema* clade together with *Ligusticum delavayi*. Group E consisted of four morphologically different species, *P. apiolens*, *P. aromaticum*, *P. rivulorum*, and *H. dentatum*, and clustered with *Levisticum* and *Vicatia* that belonged to the *Sinodielsia* clade. Group F consisted of *P. bicolor* itself and was located in the basal of Apioideae, and in the expanded ITS analysis, it clustered with *Pleurospermopsis sikkimense* and *Hymenidium miehanum* that represented the monogeneric *Pleurospermopsis* clade. Most groups were strongly supported in all analyses (SH-aLRT support > 80, Bayesian support > 90, ultrafast bootstrap support > 95), except for group E, which was weakly supported in ITS phylogeny.

**Figure 2:**
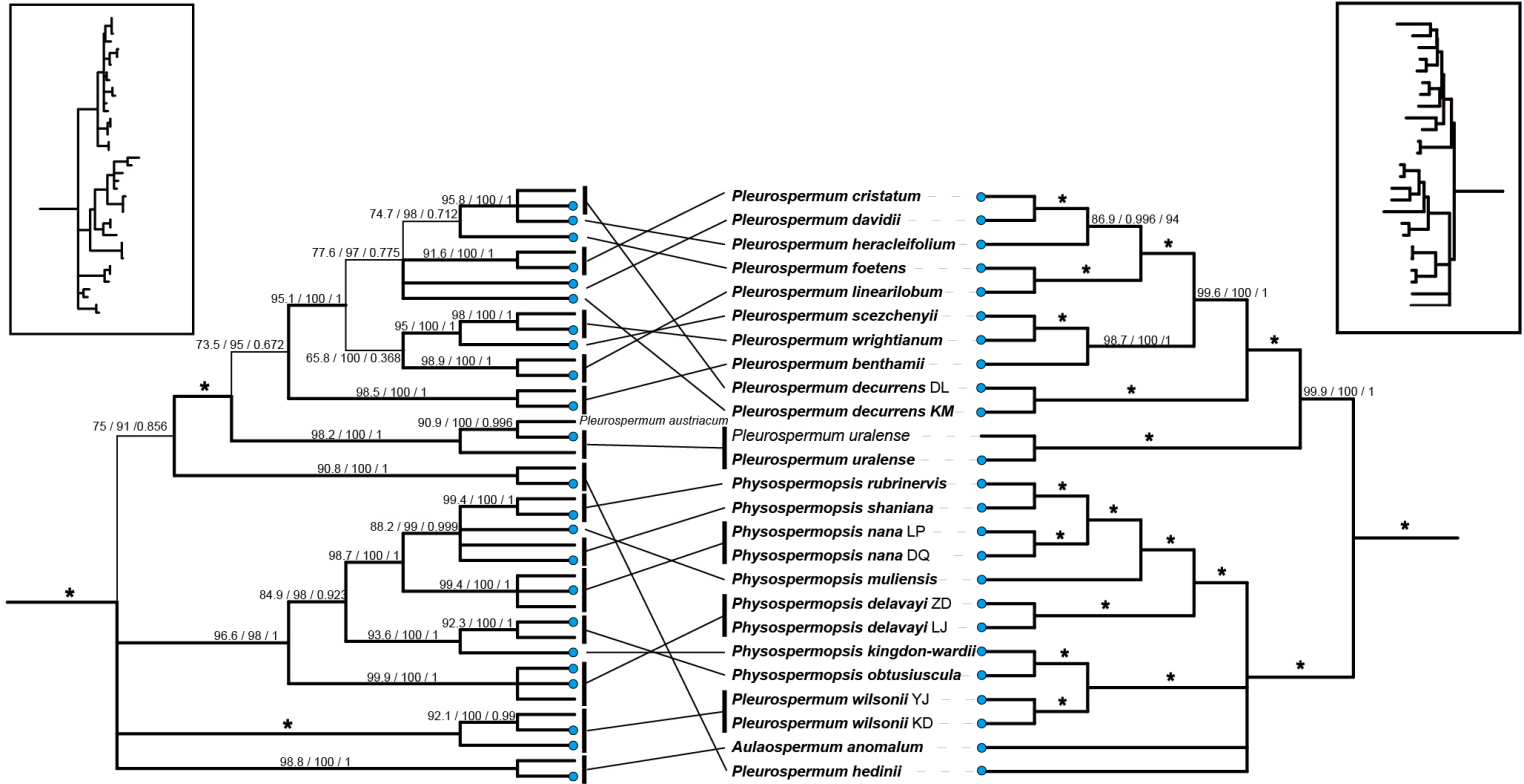
A comparison of phylogenetic relationships among species within group A (Pleurospermeae) inferred from different datasets. The left is inferred from the nrDNA (ITS1+ITS2) dataset. The right is inferred from the 79-PCG dataset from the plastomes. Branches in thinner lines indicate a low support rate. Terminal nodes marked by blue dots represent the newly sequenced ones. The overall tree topology is shown in the box on each side. * represents the best support that the maximum value is for all support rates (100/100/1).

Tree topologies yielded by ML and BI analyses were consistent in all datasets, and the support values of the nodes were much higher in the plastome dataset than in the ITS dataset. Meanwhile, strong topological incongruence between the plastid and ITS trees was noticed from the interspecific level to the intertribal level. For example, the genera *Hansenia* and *Haplosphaera*, considered as members of the East Asia clade based on ITS studies, occurred as sisters to the *Komarovia* clade and the rest to the East Asia clade. A comparison of tree topology between the plastomes and the ITS datasets is shown in **Figure 2**.

### 3.3 Plastome comparison

**Figure 3:**
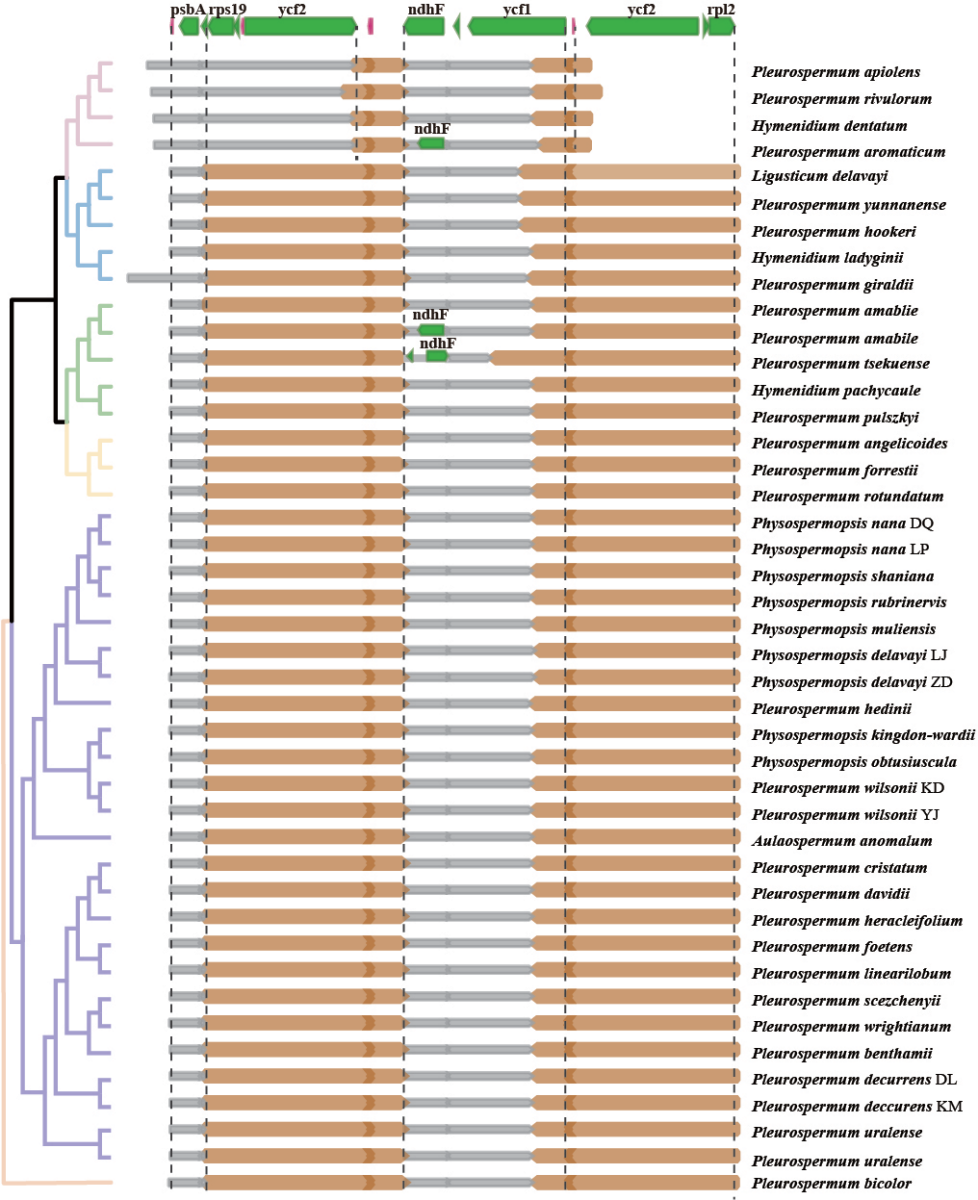
Comparison of LSC, SSC, and IR boundary regions between the 43 plastomes and their relevance to the chloroplast-based phylogeny. The boxes above represent the gene position and approximate length in alignment (green for PCG and purple for the rRNA gene). The distance (bp) of junctions to the start/end of specific genes is marked. Because of the shorter *ndhF* gene in the plastome of *P. aromaticum* and *H. amabile* and the reversion occurring in *P. tsekuense*, their genes were independently labeled in these parts and marked by red dashed boxes.

The features of the 40 newly assembled plastomes and 3 downloaded plastomes obtained from NCBI were compared on the basis of phylogenetic inference. Though morphological and ecological differences among these 36 species, their plastomes show somewhat conservatism in overall plastome structure, gene content, and gene arrangement. Junctions between regions in most plastomes are also conservative (**Figure 3**), showing a few lineage-based differences: the IRa/SSC junctions are located in the *ycf1* gene, and the IRb/SSC junctions are located between the gene *ndhF* and trnN-GGU or slightly expand into the gene *ndhF.* Meanwhile, the IRb/LSC junctions exhibit two significant types: the most common type shared by most plastomes falls into the gene *rps19* and slightly contracts to the intergenic region of the genes *rps19* and *rpl2* in some species (*P. bicolor*, *P. angelicoides*, and *P. giraldii*); the second type found in the species of group E falls into the gene *ycf2.* Indels (insertions and deletions) of significant length (over 200 bp in intergenic regions and over 10 bp in CDS) are found between taxa in different levels from individual-specific to lineage-specific (**Supplementary Table S4**). Most of these indels are deletions found in intergenic regions. The largest deletion is found in the Dali population of *P. decurrens*, which is located in the intergenic region between the *ycf4* and *cemA* genes and reaches a length of ca. 600 bp, followed by ca. 540 bp deletion shared by *Ph. nana* and *Ph. rubrinervis* located in the same region. Significant insertions are much lesser but very notable in length. The largest insertion found in the plastome of *P. giraldii* reaches a length of ca. 186,300 bp, which falls near the IRa/LSC junction, followed by an insertion of ca. 4,000 bp in *P. tsekuense* located between the *ndhF* and *trnL-UAG* genes in SSC regions. Indels within CDS are rare and mostly found in *ycf1* and *ycf2* genes, and the most notable one is a deletion of 201 bp shared by two plastomes of *P. uralense.*

**Figure 4:**
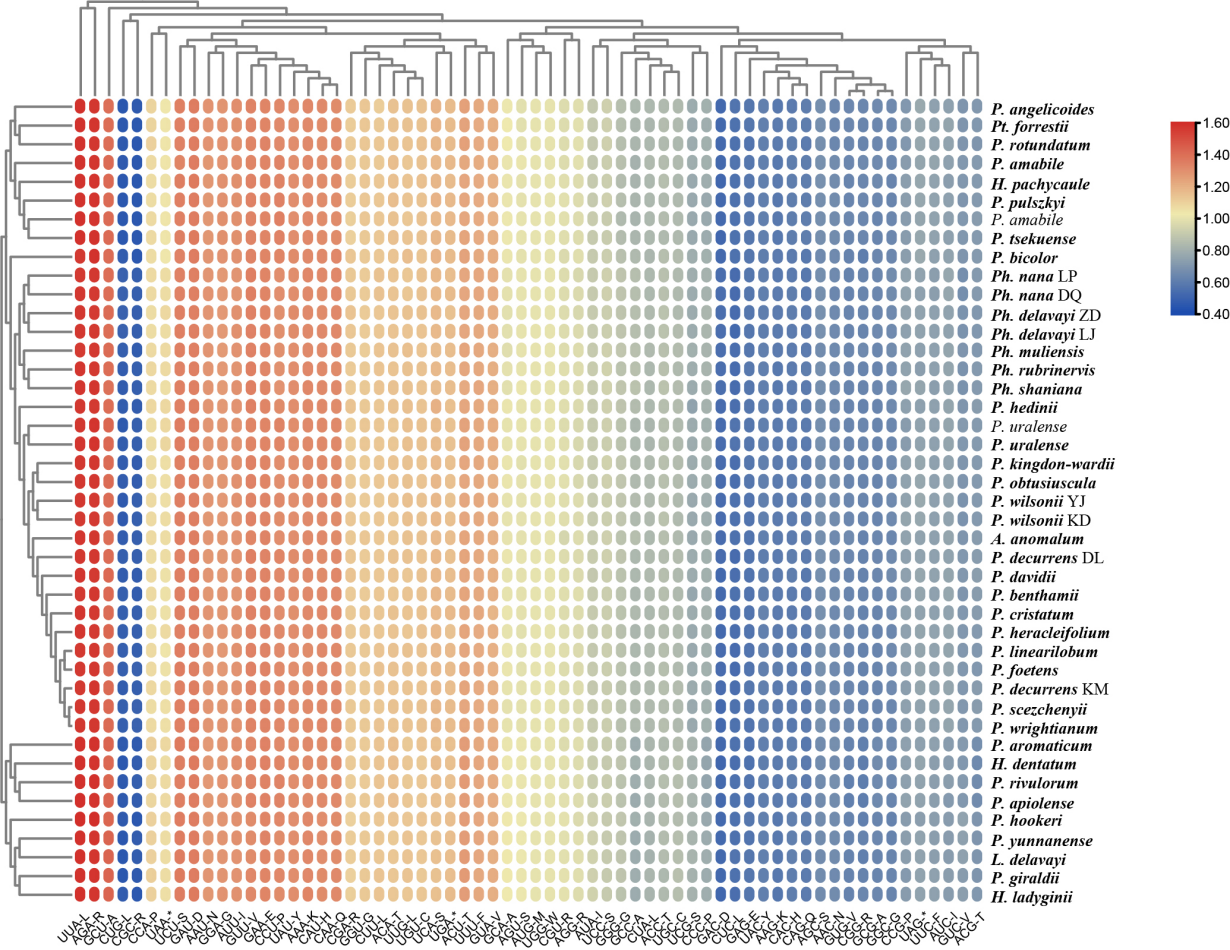
The relative synonymous codon usage (RSCU) values of all merged CDS for the 43 plastomes. The red values indicate higher RSCU values, and the blue values indicate lower RSCU values.

The frequency of codon usage was calculated based on the CDS of PCGs. Their RSCU is reported in **Figure 4** and **Supplementary Table S5**. All possible codons for all amino acids were used. Though differences in specific values exist, all lineages have the same trend in codon usage bias: the highest RSCU value is the usage of the UUA codon for leucine (1.94-2.02) followed by AGA for arginine (1.94-2.02), while the lowest is CUG for leucine (0.37-0.41) and CGC for arginine (0.37-0.40). Twenty-eight codons are used frequently with RSCU >1, and all biased codons ended with a purine (A/U). Within the 43 CDSs, the second codon positions have higher GC content (0.400-0.404) than the third codon positions (0.303-0.310). The GC contents of the total, second codon position, and third codon position are all less than 0.5, indicating the 43 plastomes tended to use A/T bases and A/T-ending codons.

**Figure 5:**
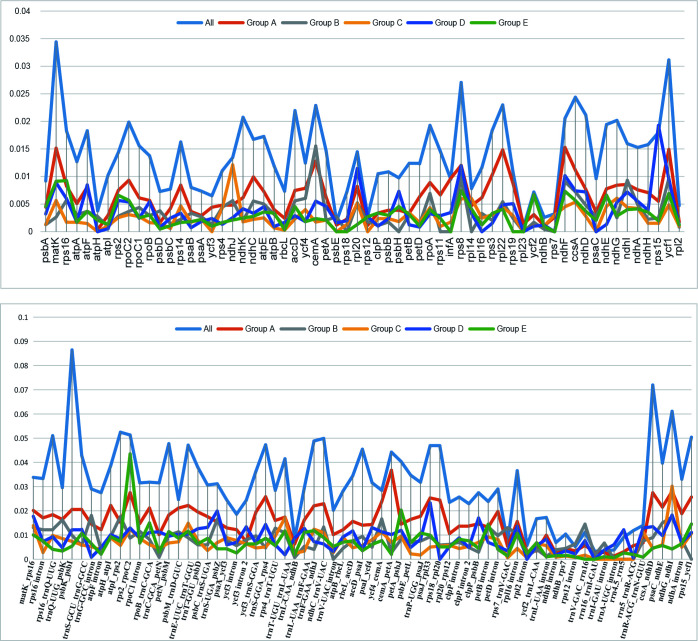
The curve of nucleotide diversity (Pi) of the 62 protein-coding regions (CDS) and intergeneric regions over 200 bp within the 43 plastomes in this study.

Nucleotide diversity (Pi) of the 43 plastomes was calculated to assess the sequence divergence level of each lineage. The overall Pi value among the 43 plastomes (0.01941) is much higher than those within each group (0.00499-0.00940). In the comparison of the Pi value of CDS among the 43 plastomes, the *matK* gene showed the highest value and *rpl23* possessed the lowest (**Supplementary Table S6**). When compared within lineages, the curve in **Figure 5** shows different trends in Pi variation between genes, which may indicate the different selection pressure and evolutionary rates of genes among lineages.

### 3.4 RNA editing events and positive selection on PCGs

The PREP suite detected 51-65 potential RNA editing sites in 35 protein-coding genes of 43 plastomes, adding up to 2,464 editing sites (**Supplementary Table S7**). All of these sites were C-to-U conversions, and over half of the sites (960 of 1,720, 32 of each plastome) are shared by all plastomes. Most RNA editing sites are situated in the second codon position (Regel and Tiling, 1838; Klotzsch et al., 1862; Handel-Mazzetti, 1933; Pimenov and Kljuykov, 1999; Пименов et al., 2011; Murrell et al., 2012; Huipeng et al., 2014; Bai et al., 2015; Murrell et al., 2015; Smith et al., 2015; Zhou et al., 2020b; Ren et al., 2022), followed by the first codon position (Zhou et al., 2008; Zhou et al., 2009; Downie et al., 2010; Zhou et al., 2020a; Zhou et al., 2021), while no sites are situated in the third codon position. Seventeen genes (*accD*, *atpA*, *atpB*, *matK*, *ndhA*, *ndhB*, *ndhD*, *ndhF*, *ndhG*, *petB*, *psaI*, *psbE*, *psbF*, *rpoB*, *rpoC1*, *rpoC2*, *rps14*) were found to have at least one editing site in all plastomes, and five (*accD*, *ndhG*, *ndhB*, *matK*, *rpoC*) possess editing sites of both kinds of codon position. Among the 35 genes tested, gene *ndhB* contained the highest number of RNA editing sites ranging from 10 to 11, followed by *rpoB* (Hoffmann, 1814; Drude, 1897-1898; Pimenov and Kljuykov, 2000b; Pimenov et al., 2000) and *ndhD* (Hoffmann, 1814; af Rantzien, 1946; Pimenov et al., 2000). Eleven kinds of conversion effects were found occurring in all plastomes, and most (8 of 11 and 81.80% of all sites) of these conversions switch the amino acids to one with higher hydrophobicity, while the most common conversion is serine to leucine (S-L) conversion (18-22 in each plastome).

**Figure 6:**
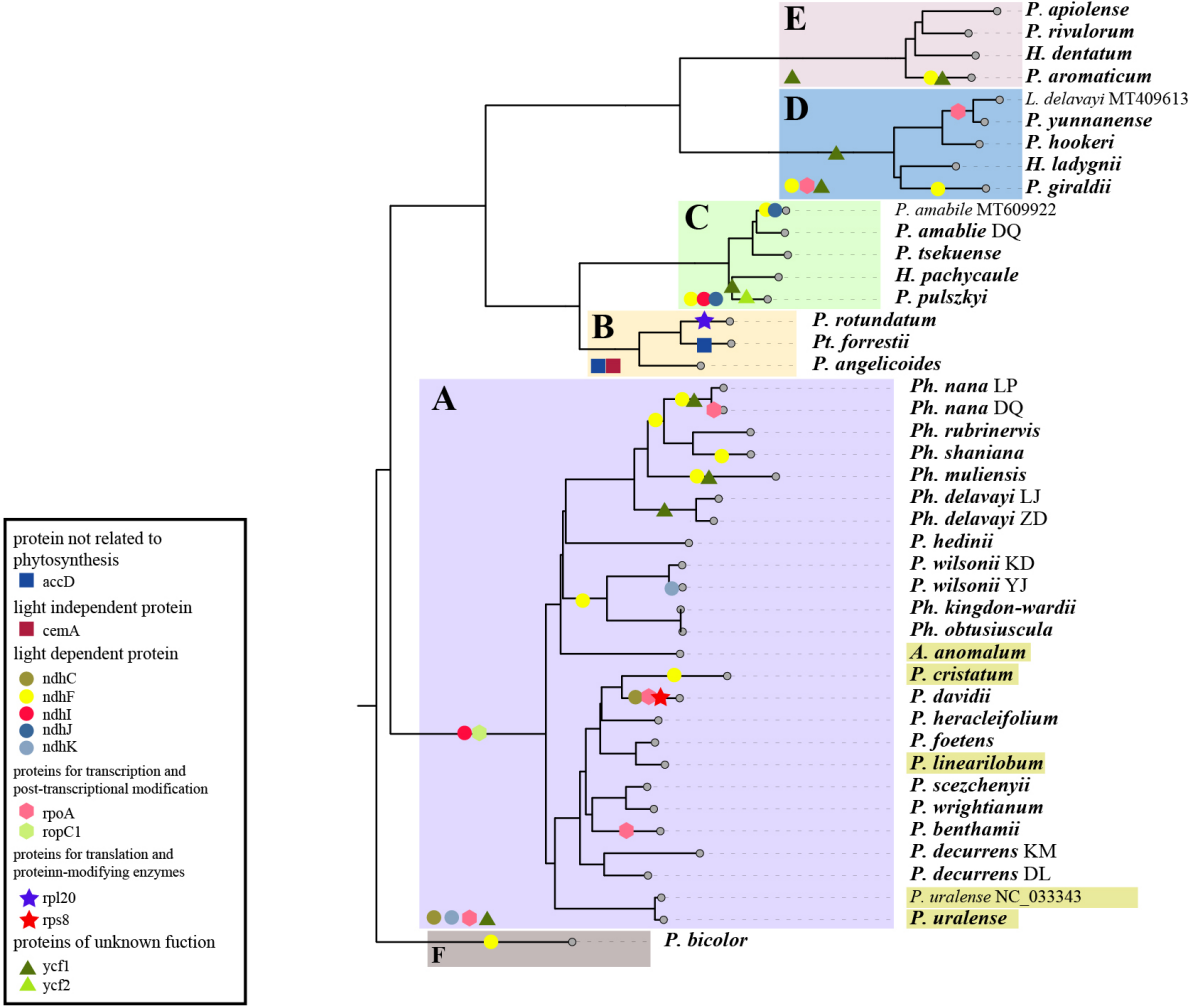
Genes detected with signals of positive selection in the 43 plastomes. The tree displayed here was pruned from the ML tree yielded from 79 plastid PCGs. Genes detected by aBSREL are marked on specific branches, and those detected by BUSTED are labeled and marked in the lower left of the colored ranges that marking the group **(A–F)**. Labels in yellow boxes indicate species distributed in relatively low altitude.

The branch-site analysis (aBSREL) and the gene-wide test (BUSTED) were applied to recognize genes undergoing positive selection, and the result differs between analyses. In all, aBSREL recognized the signal of positive selection in 12 genes (*cemA, ndhB, ndhF, ndhI, ndhJ, ndhK, rpl20, rpoA, rpoC1, rps8, ycf1* and *ycf2*). Fewer genes were detected by BUSTED, adding up to 10 (*accD, cemA, ndhC, ndnF, ndhI, ndhJ, nndhK, rpl20,rpoA* and *ycf1*). The genes detected also differ in different lineages. A total of 2-8 genes have signals of positive selection in each lineage recognized by at least one test (**Figure 6**). While no gene was recognized in all lineages by both tests, the gene *ndhF* is the one with the most recognition, followed by *ycf1*, detected in 5 and 2 and 4 and 3 groups according to aBSREL and BUSTED with 6-23 and 23-45 sites, respectively, undergoing positive selection recognized by MEME. The lineage with the highest number of genes undergoing positive selection is group A, in which 8 and 4 genes have the signature of positive selection, while in the lowest group F, the number goes to 1 and 0. Furthermore, though not all are recognized by aBSREL and BUSTED, there are 34-46 genes in each lineage that possess usually a few (Pimenov and Kljuykov, 2000a; Pan and Watson, 2005; Pimenov, 2017) sites detected by MEME.

## 4 Discussion

### 4.1 Phylogeny inference

The presence of the six lineages within *Pleurospermum* s. l. and their composition inferred by plastid phylogeny in this study are compatible with previous studies that were inferred from ITS and a few plastid fragments (Zhou et al., 2008; Zhou et al., 2009; Downie et al., 2010; Valiejo-Roman et al., 2012; Guo M. et al., 2020; Guo X-L. et al., 2020; Zhou et al., 2020a; Xu et al., 2021; Zhou et al., 2021). The support rates of these lineages are greatly improved in the plastid phylogeny of this study compared with the analyses using the ITS datasets here and also in previous studies, especially for the lineage in the *Sinodielsia* clade (group E) that was poorly supported in ITS phylogeny, thus giving strong support to the polyphyly of *Pleurospermum* s. l. and the position of these lineages in their respective clades. Among them, two lineages (group B and group F) are consistent with the recent revisions of *Pterocyclus* and *Pleurospermopsis* (Zhou et al., 2020a; Zhou et al., 2021). The rest of the lineages are more or less problematic, and most of its members were assigned to *Pleurospermum* or *Hymenidium.* Herein, we will discuss these lineages together with their morphological characteristics (**Supplementary Table S8**).

#### 4.1.1 Lineages inside Pleurospermeae

##### 4.1.1.1 Delimitation of *Pleurospermum* s. str.

After the important taxonomic revision of *Pleurospermum* and its related genera in 2000 (Pimenov and Kljuykov, 2000a), *Pleurospermum* s. str. was delimited by only two northern species, *P. uralense* and the generic type *P. austriacum*. Many of its members were transferred to *Hymenidium* and formed another complex genus. However, our phylogenetic reconstruction results showed that nine Himalayan species that were assigned to *Hymenidium* in this revision (*P. benthamii*, *P. cristatum*, *P. davidii*, *P. decurrens*, *P. foetens*, *P. heracleifolium*, *P. linearilobum*, *P. szechenyii*, and *P. wrightianum*) clustered with *P. uralense*, and together they formed a well-supported clade, which is clearly separate from other lineages in group A, namely, tribe Pleurospermeae. This is congruent with the result of a previous molecular study where six high-mountain species (*P. davidii*, *P. decurrens*, *P. foetens*, *P. linearilobum*, *P. wrightianum*, and *P. benthamii*) formed a subclade that was a sister to the two species of *Pleurospermum* s. str. Meanwhile, the generic type of *Hymenidium*, *H. brunonis*, was located far away in the *Sinodielsia* clade (Valiejo-Roman et al., 2012; Guo X-L. et al., 2020). These results indicated that the separation of the Himalayan species from *Pleurospermum* and the circumscription of *Pleurospermum* s. str. need to be reconsidered.

The *Pleurospermum* s. str. was delimited based on a set of unique characteristics of the two northern species, including inflated mericarp ribs with large secretory ducts, mesocarp separating from the mesocarp in ribs and furrows when mature (i.e., crushed mesocarp in mature fruit), flat petals, and puberulent leaf lobes (Pimenov and Kljuykov, 2000a; Valiejo-Roman et al., 2012). These two species are very closely related, and owing to the high similarity in morphology and inconspicuous geographic breaks, *P. uralense* was sometimes considered a subspecies of *P. austriacum.* Compared with the northern species, the nine Himalayan species showed a concentrated distribution in a narrower geographical range, but they were much more morphologically diverse in height, leaf, bract, and fruit characters. The most striking difference between these two taxa is the more developed bracts and bracteoles with more or less white membranous margin of the Himalayan species, especially for the alpine species. However, fruit anatomy studies show that these species share several fruit characteristics with their northern relatives, such as an obovate fruit that is more or less tuberculated, one vitta in each furrow and two in the commissure, developed fruit ribs, and most importantly, the inflated exocarp that is separate from the mesocarp in mature fruit (Huipeng et al., 2014). The last characteristic is rather unique, and it alone can set these species apart from other species in Pleurospermeae and *Pleurospermum* s. l. easily. On the basis of their close relationship shown in both plastid and ITS phylogeny and the unique synapomorphies they share, we propose a new delimitation for *Pleurospermum* s. str. to contain these nine Himalayan species along with the two northern species. The developed subequal ribs of the mericarp that are inflated, with its exocarp separate from the mesocarp when mature, are regarded as the boundary feature to distinguish this genus. All the other species in this study should therefore be excluded from *Pleurospermum.* In addition, though membranous-margined involucral bracts and bracteoles are common in the polyphyletic *Pleurospermum* s. l. and do not apply to the northern species of *Pleurospermum* s. str., they are still useful in taxonomic identification, which can help us separate the Himalayan species from the proximate genera *Aulacospermum* and *Physospermopsis*, whose bracts are leaf-like.

##### 4.1.1.2 Position of *Pleurospermum hedinii* and *Pleurospermum wilsonii*

Apart from those of *Pleurospermum* s. str. we suggested, there are still two alpine herbs that possess white membranous-margined bracts or bracteoles in Pleurospermeae: *P. hedinii* and *P. wilsonii*. Both of them fell in the other subclade that contains species of *Aulacospermum* and *Physospermopsis*. While both molecular and morphological data support that they should be excluded from *Pleurospermum* and *Hymenidium*, their proper positions are yet to be investigated. *Pleurospermum hedinii* is special in *Pleurospermum* s. l. for not only its special highly compressed appearance but also its isolation from other genera in both plastid and nuclear phylogeny. In addition to this, its relationship to other genera in Pleurospermeae varies when using different datasets in different analyses, and all of them are poorly supported. As a consequence, a new monotypic genus for *P. hedinii* should be taken into consideration. *Pleurospermum wilsonii* formed a well-supported clade with two species of *Physospermopsis* (*Ph. kingdon-wardii* and *Ph. obtusiuscula*) that are separate from other *Physospermopsis* in plastid phylogeny. However, all these *Physospermopsis* species formed a monophyletic clade in ITS phylogeny (**Figure 2**, **Supplementary Figures S2**, **S3**) that conforms to our previous study (Xu et al., 2021). Meanwhile, *P. wilsonii* clustered with *P. stellatum*, *H. huzhihaoi*, and *Trachydium roylei*, comprising a well-supported clade of high-mountain dwarf herbs that is compatible with a previous molecular study based on ITS data (Valiejo-Roman et al., 2012). These results raise questions about not only the placement of *P. wilsonii* but also the boundary between *Trachydium* and *Physospermopsis* and call for more comprehensive studies on these taxa.

#### 4.1.2 Lineages outside Pleurospermeae

##### 4.1.2.1 *Pleurospermopsis* clade

In plastid phylogeny, although *P. bicolor* fell alone at the basal of Apioideae, the expanded ITS phylogeny showed that it formed a well-supported clade with *H. miehanum* and *Pleurospermopsis sikkimense*, the generic type of *Pleurospermopsis.* Also striking in terms of developed bracts and fruit ribs, this species shares many synapomorphies with *Pleurospermopsis sikkimense* that is dissimilar to *Pleurospermum* s. str.—mostly pinnate leaves with sessile basal primary segments, compact umbellets, 1-3 lobed bracteoles, herbaceous bract texture, narrowly oblong and glabrous fruit, and 3/6 vittae in each furrow/in the commissure. Therefore, although the plastome data of *Pleurospermopsis sikkimense* were not included in our study, *P. bicolor* has already shown its isolation from other lineages as well as a special location in Apioideae, and we support its attribution to *Pleurospermopsis.*

##### 4.1.2.2 *Komarovia* clade

Aligned here are *P. angelicoides*, *Pt. forrestii*, and *P. rotundatum*, which formed a monophyletic lineage (group B) nested with *Chuanminshen* and *Changium* that represent the *Komarovia* clade. Morphologically, their oblong fruit with a much larger length–width ratio of these species is already shown in *Pleurospermum* s. l. As a consequence, segregation of these species from *Pleurospermum* was proposed very early along with the establishment of *Pterocyclus* based on *P. angelicoides* in 1862 and the later transfer of *Pt. forrestii*, *P. rotundatum*, and *P. rivulorum* to *Pterocyclus* in succession (Klotzsch et al., 1862; Handel-Mazzetti, 1933; Pimenov and Kljuykov, 1999; Pimenov and Kljuykov, 2000a). Recent molecular phylogenetic studies have verified the close relationship of these species and recognized the monophyly of this genus based on ITS data (Zhou et al., 2021), but *P. rivulorum* was excluded as it fell far away in the *Sinodielsia* clade, which is consistent with our results.

##### 4.1.2.3 East Asia clade

Phylogenetic reconstructions based on the ITS dataset have revealed that there are many species related to *Pleurospermum* s. l. (species under the name of *Hymenidium*, *Hymenolaena*, *Keraymonia*, *Physospermopsis*, and *Trachydium*) nested in this major clade, and most of them are alpine herbs with restricted distribution, of which four species that comprise group C (*P. amabile*, *P. pulszkyi*, *P. tsekuense*, and *H. pachycaule*) are the typical species. These species are distributed in higher altitudes (over 4,000 m) and possess showy bracts and bracteoles that are almost completely membranous. The most famous one, *P. amabile*, has been studied extensively in many aspects; hence, its segregation from *Pleurospermum* had been already identified at the molecular aspect (Valiejo-Roman et al., 2012; Guo M. et al., 2020) and gained support from a fruit anatomical study (Huipeng et al., 2014). Unlike *P. amabile*, the other four species are less known, which may be due to their less showy dwarf plants or restricted distribution (e.g., *H. pachycaule* found only in Lianhua Shan). As a consequence, although their close relationship was suggested in Pimenov’s revision of *Hymenidium* (Pimenov and Kljuykov, 2000b), not until now has it been supported at the molecular level. Several anatomical characteristics such as smooth mericarp, 3/6 vittae in each furrow/in the commissure, prominent calyx teeth, and almost completely membranous bracts and bracteoles distinguish this group from *Pleurospermum* s. str. and also indicate its close relationship with *Hymenolaena*, another genus of Himalayan species restored from *Pleurospermum*. However, though these two taxa showed a rather close relationship in ITS phylogeny, they failed to form a monophyletic clade (Supplementary Data S3), which conforms to their attribution to different genera in Pimenov’s revision. Therefore, as a distinguishable monophyletic group that contains no type species and yet no genus known to place in, we suppose a new genus to be established.

##### 4.1.2.4 *Acronema* clade

Group E here is comprised of three species of *Pleurospermum* s. l. (*P. giraldii*, *P. hookeri*, and *P. yunnanense*), one lately published in *Hymenidium* (*H. ladyginii*) (Пименов et al., 2011), and one traditionally assigned to *Ligusticum* (*L. delavayi*). This is a group of alpine herbs that are morphologically diverse, differing in plant size, leaf shape, bracts, bracteoles, etc. The close relationships of these species have been indicated since their attribution to *Hymenidium* (Pimenov and Kljuykov, 2000b) and verified later by several molecular studies (Zhou et al., 2008; Zhou et al., 2009; Пименов et al., 2011; Valiejo-Roman et al., 2012; Bai et al., 2015; Ren et al., 2022). While it is clear that the proper placement of group D is neither *Hymenidium* nor *Pleurospermum*, the estimated phylogeny based on ITS indicated its close relationship to *Rupiphila tachiroei* (Franch. & Sav.) Pimenov & Lavrova and *Tilingia ajanensis* Regel & Tiling, the generic type of *Rupiphila* and *Tilingia* Regel & Tiling. Both genera were once regarded as a synonym of the complex traditional *Ligusticum* for their uncertain circumscription, and their separation was accompanied by the delimitation of *Ligusticum* s. str. by only two species in the *Acronema* clade on the basis of ITS data (Zhou et al., 2020b; Ren et al., 2022). *Tilingia* was then proposed to receive these taxa for it has priority. *Tilingia* was established based on its solitary vittae in each furrow and prominent calyx teeth (Regel and Tiling, 1838), which are not unique in the *Acronema* clade. After careful examination of the specimens of these species, we found that the morphological synapomorphies can be summarized by prominent calyx teeth, oblong fruit with prominent or winged ribs that are subequal, and bracts and bracteoles from linear to lanceolate with more or less membranous margin. Though this character set is distinguishable within this clade, the prominent differences in vittae number among *T. ajanensis* (1/2), *Rupiphila tairoei* (1/2), and species of group C (1-4/5-6 in *H. ladyginii*, 3/6 in other species), as well as multiformity in leaf morphology, are notable. In addition, the existing molecular evidence is barely based on ITS sequence and thus rather doubtful.

##### 4.1.2.5 *Sinodielsia* clade

Group E that belongs here is rather tricky as it contains four morphologically dissimilar species, *P. aromaticum*, *P. apiolens*, *P. rivulorum*, and *Hymenidium dentatum*, that are attributed to different genera (*P. aromaticum* to *Oreocomopsis*, *P. apiolens* and *H. dentatum* to *Hymenidium*, and *P. rivulorum* to *Pterocyclus*) by Pimenov (Pimenov and Kljuykov, 2000a; Pimenov and Kljuykov, 2000b). Previous studies found that the nomenclatural type of *Hymenidium*, *H. brunonis*, may also belong to this lineage (Valiejo-Roman et al., 2012; Guo X-L. et al., 2020), which disapproved the separation of the southern group of *Pleurospermum* s. l. as the genus *Hymenidium* at the molecular aspect, and this is supported in our ITS phylogeny. The shared characteristics of these alpine herbs are their membranous margin of bracts and bracteoles and oblong to elliptic fruit with developed ribs, which account for their position in *Pleurospermum* s. l., but they can be separated from *Pleurospermum* s. str. easily by the key characteristics in fruit anatomy. However, they are much dissimilar in plant size, leaf shape, and some fruit anatomy characters like vittae counts (**Supplementary Figure S8**). Unfortunately, while the present circumscription of *Hymenidium* is clearly not suitable for this group, the primal concept of *Hymenidium* that is established on the basis of plane seed face cannot separate them from other species in the *Sinodielsia* clade. Worse still, considering the poor support rate of this group in ITS phylogeny, whether they should be included in *Hymenidium* is still dubious. In addition to this, the name *Sinodielsia* clade was found controversial because of the misidentification of *Tongoloa zhongdianensis* to *S. delavayi* in its establishment (Gou et al., 2021). Far from the generic type of *Tongoloa* in the East Asia clade, the placement of *T. zhongdianensis* remains dubious. Therefore, the “*Hymenidium* clade” would be a proper replacement given the possible attribution of *P. rivulorum* to *Hymenidium.*

#### 4.1.4 Topological incongruence between nuclear and plastid datasets

The usage of data from complete plastomes has greatly improved the phylogenetic resolution especially in lower levels and provided us with more distinct and reliable intergeneric and interspecific relationships for the species in *Pleurospermum* s. l. when compared with previous studies. However, even if we ignore the less credible nodes from ITS trees, topological incongruence can be noticed between the nuclear and plastome datasets, making it difficult to understand the interspecific relationships within these taxa. For example, in the *Pleurospermum* s. str. we proposed, two samples of *P. decurrens* clustered and have more basal position which is next to *P. uralense* in plastid phylogeny, but they fell apart in ITS phylogeny, and one of them clustered with *P. heracleifolium* that was more distally located in plastid phylogeny (**Figure 2**). Such incongruences are also noticed in *Physospermopsis* and group D. Incongruence between the plastid and nuclear phylogenetic tree in Apiaceae has been reported several times and hypothesized as either incomplete lineage sorting (Wen et al., 2020), hybridization/introgression (Calvino et al., 2008; Yi et al., 2015; Fernández et al., 2017; Wen et al., 2021), or both. In the case of *P. decurrens*, hybridization seems to be a proper account. The species of *Pleurospermum* s. str. produce typical inflorescence of Apioideae, which possess entomophilous characters, such as their flat umbellule that serve as platforms and the arresting petal-like bracts and surrounding bracteoles. Thus, cross-pollination and interbreeding are encouraged in these taxa. Hybridization events are frequently found in Apioideae, which may collectively reflect the rapid radiation of its members along with the possible incomplete lineage sorting (Wen et al., 2021). The existence of incongruence between the two datasets also means that, though providing abundant informative sites and yielding phylogeny with high resolution, solely plastome data may not be sufficient for the systematic study of this genus (or tribe). Unfortunately, the current nuclear-based phylogeny about this genus is all barely based on ITS data and shows low resolution. Therefore, more nuclear data would be essential to investigate the convincing interspecific relationships of these taxa.

### 4.2 The diversity of plastome characteristics

From the *Pleurospermopsis* clade basal in Apioideae to the distal *Sinodielsia* clade, all lineages of *Pleurospermum* s. l. and related genera we studied show great conservatism in plastome structure and gene content. All studied plastomes share a typical quadripartite structure, including a pair of IRs separated by the LSC and SSC regions, with 79 unique PCGs, 30 tRNA, and 4 rRNA genes that are arranged mostly in identical order. Generally, the most typical reason for variation in size and rearrangements of the plastome is the expansion or contraction of IRs into or out of adjacent single-copy regions (Downie and Jansen, 2015; Weng et al., 2017). Therefore, as IR borders are mostly similar in group A to group D that are located in the basal part of Apioideae, their plastomes are also similar in size especially in comparison within lineages, varying within a range of 5% in length. In group E, the most distally branching group of the six groups, where a contractive type of IR border is found (**Figure 3**), their much smaller plastome size is in line with expectations. These also cohered with what Wen et al. (2021) found in the backbone phylogeny and evolution of Apioideae based on plastome data: contractive IRb/LSC junction types occurred in some distally branching clades, while the expansive types were shared among the basally branching lineages. It may reflect the less conserved plastome of distally branching clades than the relative basally branching clades.

On the other hand, several plastomes with extraordinary size can be noticed, and here comes another important factor affecting genome size, insertion. Large insertion contributes to the three largest plastomes (*P. giraldii*, *P. tsekuense*, and *P. bicolor*) in our study. The insertion found in the largest plastome of *P. giraldii* reaches 16,300 bp and is located beside the IRa/LSC junction. In *P. tsekuense*, an insertion of ca. 4,000 bp occurs in SSC. Together with ca. 2,200 bp expansion of IR regions at the IRa/SSC border, this insertion contributes to its second largest genome size. Though found in different locations, BLAST analysis revealed that these large inserted sequences have possible origin from the mitochondria genome and some genes in the case of *P. giraldii*. Transfer of foreign DNA in the plastome is a rare event (Smith, 2014), and only a few cases of mt-to-pt DNA transfer in flowering plants have been confirmed (Iorizzo et al., 2012a; Iorizzo et al., 2012b; Ma et al., 2015; Spooner et al., 2017). Due to its rarity, this type of insertion would be useful to trace ancestry. However, all these large insertions found in our study are more species-specific, unique among the studied plastomes, and thus, more suitable for their identification. However, some significant indels with a smaller size (over 200 bp) show more lineage specificity (**Supplementary Table S4**), which may be helpful in identification. These variances are also more or less reflected by GC content. In group A, for example, where most plastomes exhibit tidy overall GC content of 37.9, indels up to 90 bp can be found in those plastomes with slightly higher GC content of 38. Likewise, IR contraction in group E and insertion in the largest three plastomes were also reflected by their “abnormal” GC content of corresponding regions. Therefore, this parameter can be an indicator of structural variation. The frequency of codon usage is an important evolutionary feature in the genome and is mainly influenced by mutation pressure on DNA sequences and natural selection (Mitreva et al., 2006). Therefore, it provides useful information for studying molecular evolution. All 43 plastomes studied show similar patterns of frequently used codons, with a strong bias toward A/T at the third codon position. It is consistent with previous reports from many other chloroplast genomes (Yang et al., 2018; Gu et al., 2019). Still, the RSCU value slightly differs between lineages, which is clearly reflected in integrated data, such as the noticeable lower codon bias index (CBI) and the higher GC content in all species of group A than others.

### 4.3 The evolution of protein-coding genes

As a group of mostly alpine herbs, the resemblance morphology of the members of *Pleurospermum* s. l. brings several problems to taxonomists. Plants that live in such regions are challenged by harsh conditions with increasing altitude, such as strong solar radiation, low temperature, wind velocity, aridity, shorter vegetation period, and the rising number of weather-related extreme events in highlands (Peng et al., 2011), which limit their plant growth and distribution. Similar stress and stimuli in alpine or subalpine habitats may lead to a parallel strategy for survival, yielding a semblable appearance in alpine plants, and it is legitimate to hypothesize that such effort would also be reflected in plastid genomes, as they encode numerous proteins for photometabolic pathways that are essential for their survival (Wicke et al., 2011).

The plastid genes we detected with signals of positive selection among these species are involved in different processes, including photosynthesis (*accD*, *ndhC*, *ndhF*, *ndhI*, *ndhJ*, and *ndhK*), lipid acid synthesis (*accD*), transcription (*rpoA* and *rpoC1*), translation (*rpl20* and *rps8*), and unknown function (*ycf1* and *ycf2*). Among them, *ndh* genes are the most common and *ndhF* is positively selected with the highest number of nodes. This is a group of genes encoding subunits of the thylakoid NDH (NADH dehydrogenase-like) complex that mediate the cyclic electron flow (CEF). They help with the stabilization of the NDH complex, especially under strong light conditions, and further affect the photosynthetic efficiency (Martin and Sabater, 2010; Wicke et al., 2011). Thus, these genes might play important roles for these species to maintain photosynthesis in the adaption of strong solar radiation in alpine areas. Another gene also detected in the five lineages is *ycf1*, which is known to evolve rapidly in most land plants, reflected by numerous sites detected by MEME, and may undergo pseudogenization. This gene was found expressed in fruits and thought to be essential for plant cell survival (Drescher et al., 2000). However, its precise function remains to be determined. Other genes detected are more specific in different levels of taxa, such as *accD*, *cemA*, and *rpl20* detected only in group B, *rps8* in *P. davidii*, and *ndhK* in the Yajiang population of *P. wilsonii*. The pattern of these detected genes shows no significant association with neither morphological characteristics nor altitudes or other factors we considered, and we supposed that they might be related to more specific environmental factors of populations, which implies the complexity of these species in alpine adaptation with multiple mechanisms involved underlying their resemblance appearance.

RNA editing is a posttranscriptional modification process that occurs in the chloroplast and mitochondrial genomes identified in all groups of land plants. Primordially acting as error correctors to maintain the function of proteins for the early land plants facing strong solar radiation, it also acts in regulatory functions and produces variants of proteins to adapt to different physiological needs (Tseng et al., 2013; He et al., 2016; Chen et al., 2017). Therefore, patterns of RNA editing sites may also reflect the adaptation of these species in another aspect.

Putative editing sites in the plastomes of all lineages of *Pleurospermum* s. l. are relatively conservative and conform to those observed in other Apioideae species (Ren et al., 2020; Liu et al., 2022) as well as in most angiosperms (Tseng et al., 2013; He et al., 2016). Comparing between plastomes, these sites can be roughly divided into three types: 1) shared by all 43 plastomes, consisting of 71.55% of all editing events; 2) lost in the distal taxa of different levels as back mutation of C to T occurs, making up 27.8% of all editing events; and 3) occurred in a specific taxon. The dominance of the first type reflects the conservatism of the RNA editing system among species of *Pleurospermum* s. l., while the second indicates the slow but ongoing loss of editing sites along with evolution. He et al. propose the diverse evolutionary paths of editing sites depending on their influence on protein functions: In essential genes, editing sites disappear during plant evolution due to reverse mutation at the DNA level; in non-essential genes, loss of editing sites would be random and slow, resulting in more sites preserved (He et al., 2016). The gene with the highest number of RNA editing sites detected herein is *ndhB*, in which 10 of 11 editing sites are shared by all plastomes. A high number of editing sites in *ndh* genes are also observed in other land plants reported (Maier et al., 1995; He et al., 2016). Though closely related to photosynthesis as we demonstrated above, *ndh* genes have been proven dispensable in optimal growth for some plants and partially or completely lost in some parasite plants (Burrows et al., 1998; Funk et al., 2007; Chris Blazier et al., 2011). Therefore, the high number of editing sites kept in these genes can be explained as they are not essential. However, in the cases of *Pleurospermum* s. l., the extremely high conservatism of the editing sites of *ndhB* indicates another possibility that they were retained by purifying selection. Decay in the editing efficiency of *ndh* genes has been proven to impair photosynthetic efficiency but improves stress tolerance under harmful conditions (Garcia-Andrade et al., 2013). Therefore, these highly conservative editing sites of *ndhB* may act as a switch for these alpine Apiaceae to initiatively or passively lower the photosynthetic efficiency for enhanced stress tolerance facing the unstable environment. Other than *ndhB*, editing sites are fewer and more variable in other *ndh* genes, and four of the five genes detected by the positive selection test above (except for *ndhF*) even possess no editing sites, indicating the diverse evolutionary directions within this gene family. Similar to the analyses of positive selection, genes with variable editing sites among lineages are associated with various functions including biosynthesis, transcription, and translation, which at the posttranscriptional level indicate the diverse effort for these plants in alpine adaptation.

## 5 Conclusion

By using the plastome data of 36 species from *Pleurospermum* s. l. and related genera, we obtained a backbone phylogeny for *Pleurospermum* s. l. in the Apioideae background with improved resolution. The results confirmed the polyphyly of *Pleurospermum* s. l. as well as the positions of its six lineages that fell in Pleurospermeae, *Komarovia* clade, East Asia clade, *Acronema* clade, *Sinodielsia* clade, and *Pleurospermopsis* clade, respectively. Combining the strong support from both plastid and nuclear phylogenetic analyses with morphological data, we propose an expanded circumscription for *Pleurospermum* s. str. to contain nine Himalayan species that clustered with *P. uralense* in Pleurospermeae. The boundary features of this genus were defined as the ovoid fruit with developed ribs that are inflated, with its exocarp separate from the mesocarp when mature. In addition, involucral bracts and bracteoles with more or less white membranous margins are also regarded as important diagnostic characteristics. The other two species (*P. wilsonii* and *P. hedinii*) in Pleurospermeae and the species in the other five lineages are supposed to be excluded from *Pleurospermum*. We support the latest revision of *Pterocyclus* (group B) (Zhou et al., 2021) and *Pleurospermopsis* (group F) (Zhou et al., 2020a), while the remaining three lineages remain unresolved and need further investigation. On the basis of phylogenetic inference, comparative analyses found that these plastomes are conserved in terms of genome size, genome structure, IR borders, codon bias, and RNA editing sites among taxa. Still, certain differences were found between taxa of different levels, especially for group E that clustered in the distally branching *Sinodielsia* clade. In addition, positive selection analyses detected different PCGs among these lineages which may indicate their underlying unique evolutionary history in alpine adaptation and the diverse strategy for survival beneath their similar morphology. Overall, our study proposed a renewed delimitation for *Pleurospermum* s. str. and provided a framework for future taxonomic and phylogenetic studies of the problematic taxa within the polyphyletic *Pleurospermum* s. l.

## Data availability statement

The datasets presented in this study can be found in online repositories. The names of the repository/repositories and accession number(s) can be found in the article/**Supplementary Material**.

## Author contributions

CP, X-LG and X-JH conceived and designed the experiments. CP and X-LG analyzed the sequence data and drafted the manuscript. CP and X-JH participated in the data analysis and manuscript drafting. CP, X-LG, X-JH and S-DZ revised the manuscript. All authors contributed to the article and approved the submitted version.

## Supplementary material

Supplementary Figure 1 The gene maps of the six lineages of *Pleurospermum* s. l. recognized.

Supplementary Figure 2 Phylogenetic relationships inferred from nuclear dataset of 86 species that adopted in plastid phylogeny.

Supplementary Figure 3 Phylogenetic relationships inferred from the expanded nuclear dataset of 208 sets of ITS1+ITS2 data from 169 species all over the Apioideae.

Supplementary Figure 4 alignment of the 43 plastomes from *Pleurospermum* s. l. and related genera by mVISTA.
