# Supplementary figures and images for: Backbone phylogeny and adaptive evolution of Pleurospermum s. l.: New insights from phylogenomic analyses of complete plastome data
Source: Front Plant Sci. 2023 Mar 30;14:1148303. doi: 10.3389/fpls.2023.1148303 (PMC10101341; doi:10.3389/fpls.2023.1148303)

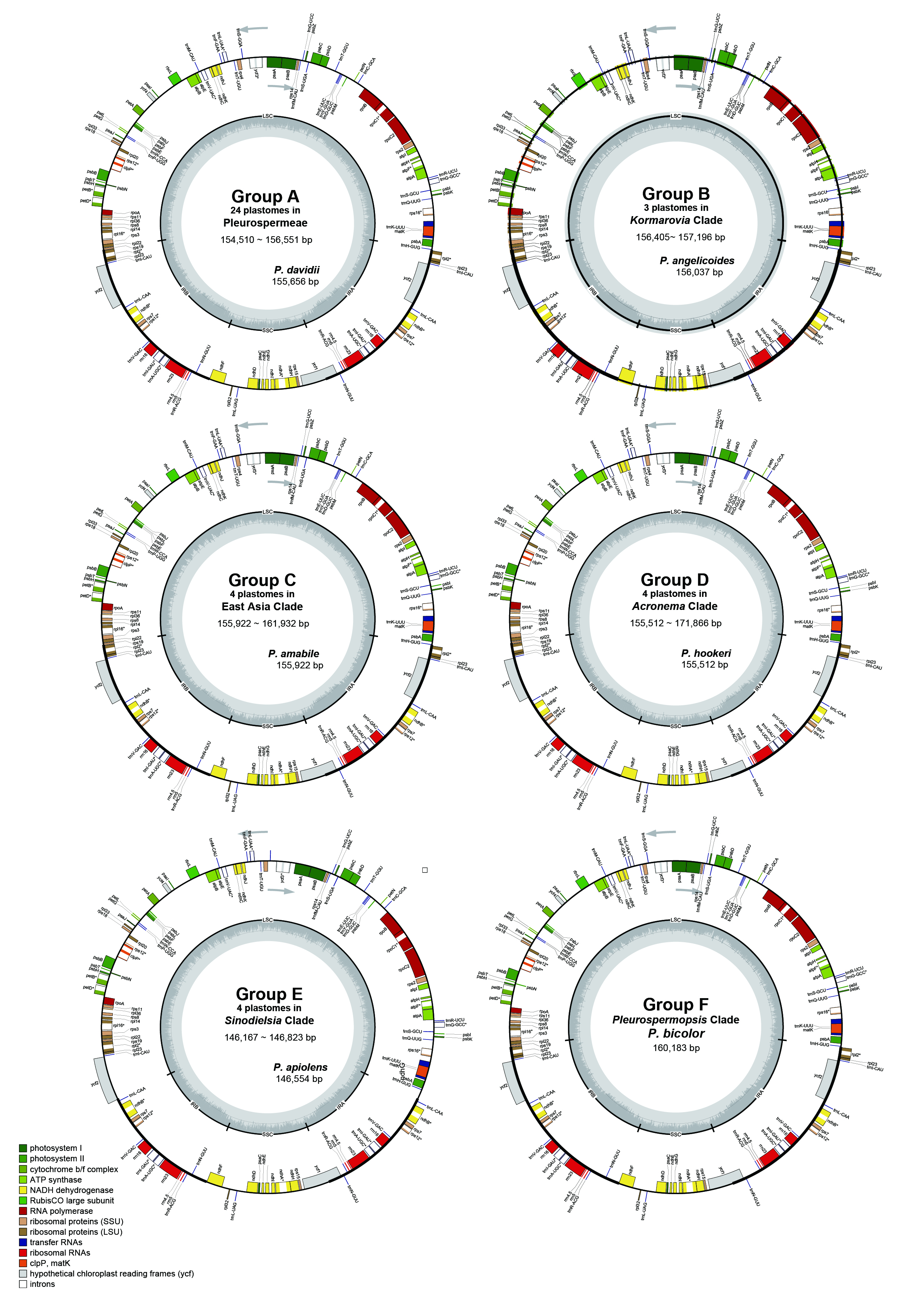

Supplement: Supplementary Figure 1 — The gene maps of the six lineages of Pleurospermum s. l. recognized. [file Image_1.jpg]

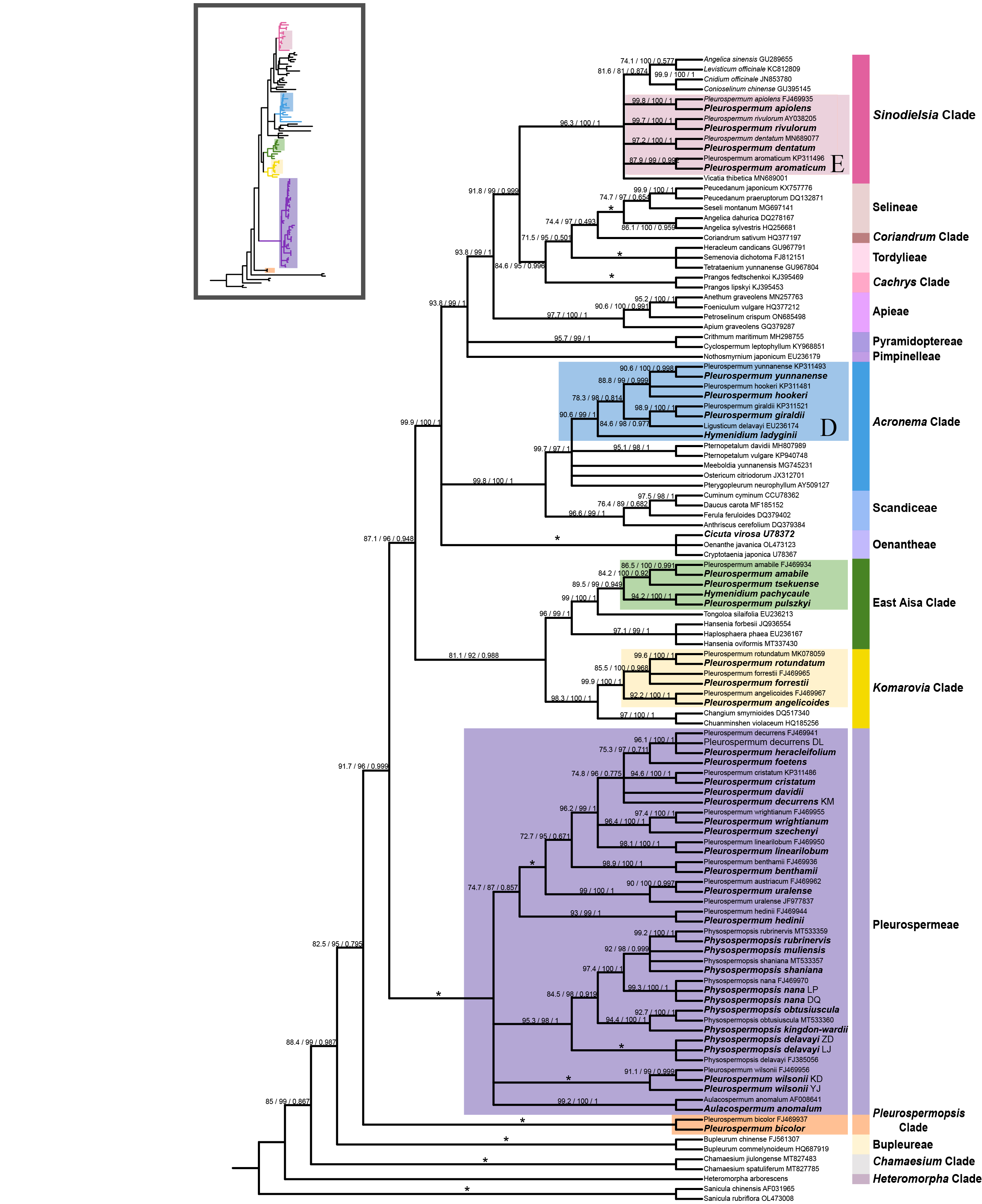

Supplement: Supplementary Figure 2 — Phylogenetic relationships inferred from nuclear dataset of 86 species that adopted in plastid phylogeny. [file Image_2.jpeg]

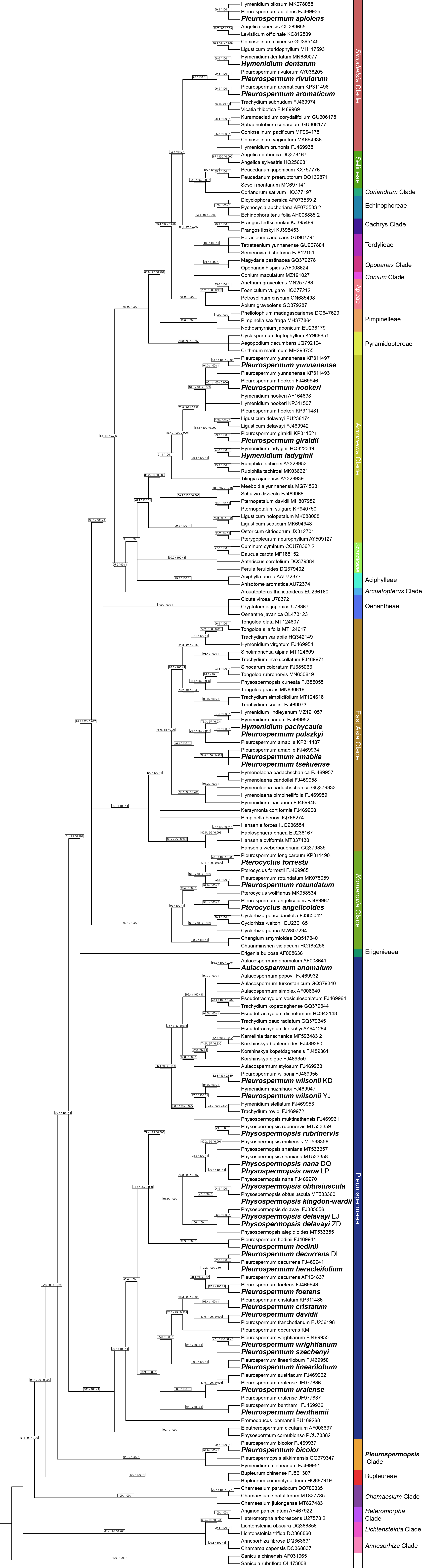

Supplement: Supplementary Figure 3 — Phylogenetic relationships inferred from the expanded nuclear dataset of 208 sets of ITS1+ITS2 data from 169 species all over the Apioideae. [file Image_3.jpeg]

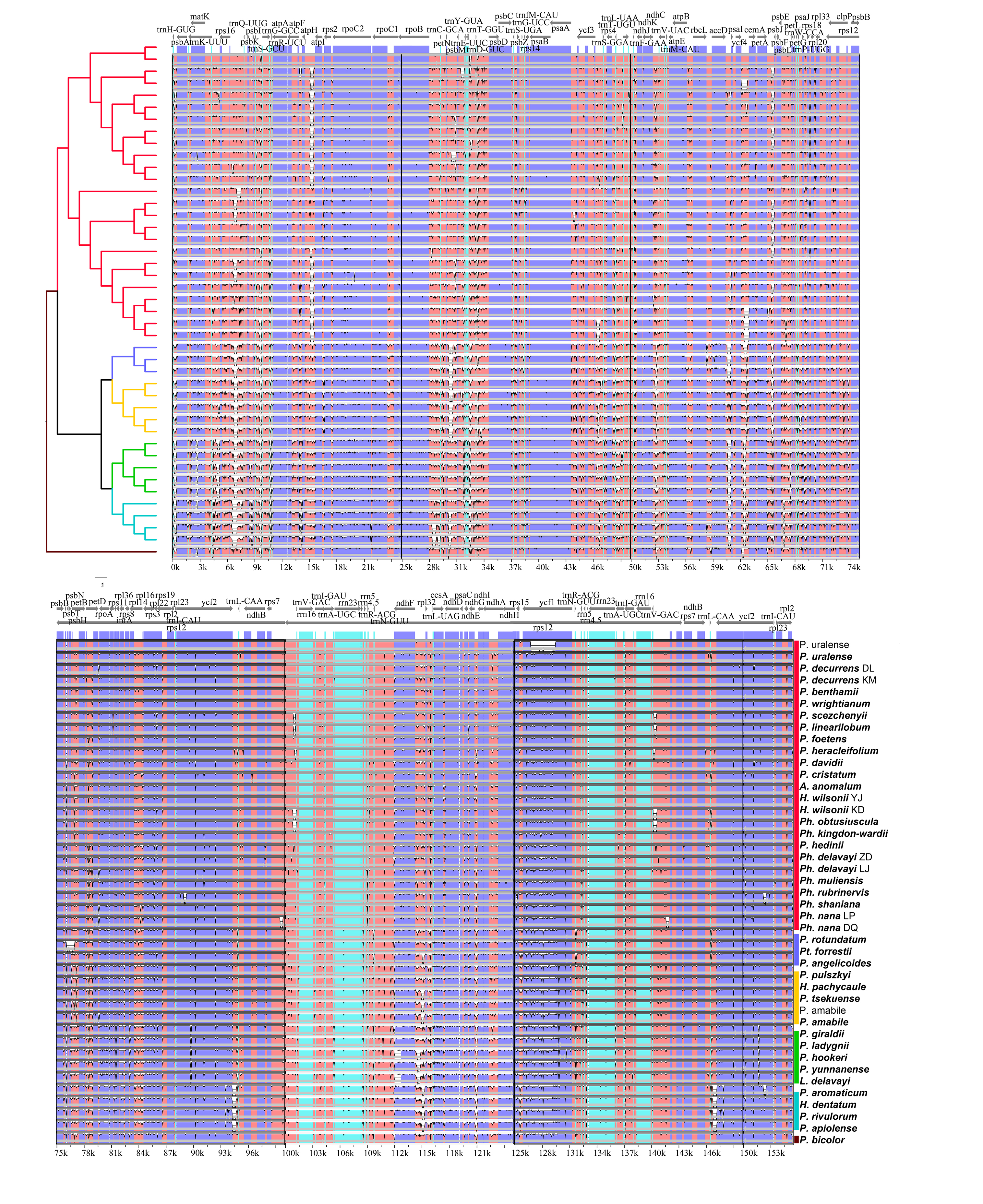

Supplement: Supplementary Figure 4 — alignment of the 43 plastomes from Pleurospermum s. l. and related genera by mVISTA. [file Image_4.jpeg]

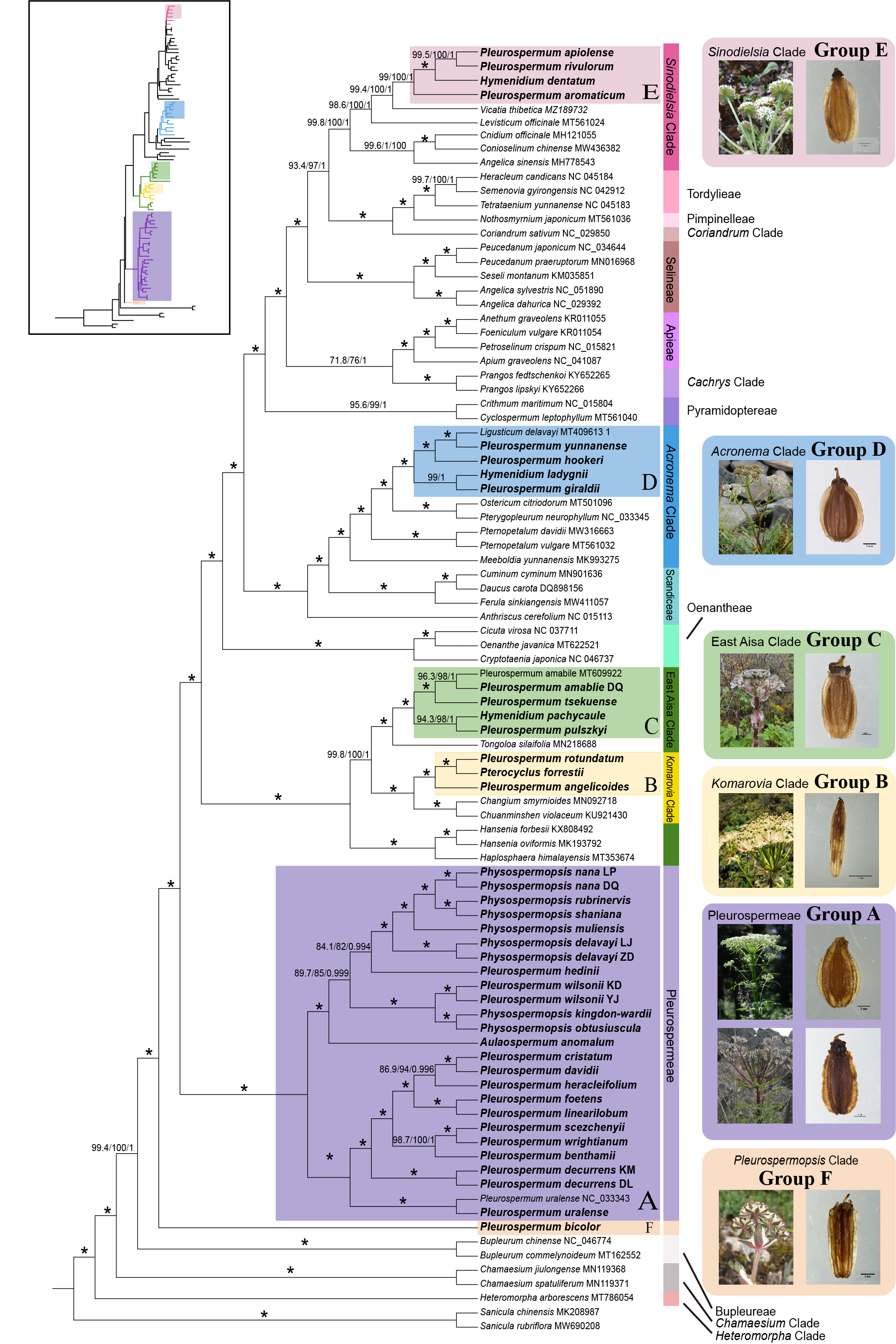

Supplement: Supplementary file 5 [file Image_5.jpeg]
